# Emerging Low Detection Limit of Optically Activated Gas Sensors Based on 2D and Hybrid Nanostructures

**DOI:** 10.3390/nano14181521

**Published:** 2024-09-19

**Authors:** Ambali Alade Odebowale, Amer Abdulghani, Andergachew Mekonnen Berhe, Dinelka Somaweera, Sanjida Akter, Salah Abdo, Khalil As’ham, Reza Masoudian Saadabad, Toan T. Tran, David P. Bishop, Alexander S. Solntsev, Andrey E. Miroshnichenko, Haroldo T. Hattori

**Affiliations:** 1 School of Engineering and Technology, The University of New South Wales at Canberra, Campbell, ACT 2612, Australia; a.odebowale@unsw.edu.au (A.A.O.); a.abdulghani@unsw.edu.au (A.A.); a.berhe@unsw.edu.au (A.M.B.); d.liyadde_gedara@unsw.edu.au (D.S.); sanjida.akter@unsw.edu.au (S.A.); s.abdo@unsw.edu.au (S.A.); k.asham@unsw.edu.au (K.A.); andrey.miroshnichenko@unsw.edu.au (A.E.M.); 2 School of Mathematical and Physical Sciences, University of Technology Sydney, Ultimo, NSW 2007, Australia; reza.masoudiansaadabad@uts.edu.au (R.M.S.); trongtoan.tran@uts.edu.au (T.T.T.); david.bishop@uts.edu.au (D.P.B.); alexander.solntsev@uts.edu.au (A.S.S.)

**Keywords:** limit of detection, response time, selectivity, sensitivity, UV irradiation

## Abstract

Gas sensing is essential for detecting and measuring gas concentrations across various environments, with applications in environmental monitoring, industrial safety, and healthcare. The integration of two-dimensional (2D) materials, organic materials, and metal oxides has significantly advanced gas sensor technology, enhancing its sensitivity, selectivity, and response times at room temperature. This review examines the progress in optically activated gas sensors, with emphasis on 2D materials, metal oxides, and organic materials, due to limited studies on their use in optically activated gas sensors, in contrast to other traditional gas-sensing technologies. We detail the unique properties of these materials and their impact on improving the figures of merit (FoMs) of gas sensors. Transition metal dichalcogenides (TMDCs), with their high surface-to-volume ratio and tunable band gap, show exceptional performance in gas detection, especially when activated by UV light. Graphene-based sensors also demonstrate high sensitivity and low detection limits, making them suitable for various applications. Although organic materials and hybrid structures, such as metal–organic frameworks (MoFs) and conducting polymers, face challenges related to stability and sensitivity at room temperature, they hold potential for future advancements. Optically activated gas sensors incorporating metal oxides benefit from photoactive nanomaterials and UV irradiation, further enhancing their performance. This review highlights the potential of the advanced materials in developing the next generation of gas sensors, addressing current research gaps and paving the way for future innovations.

## 1. Introduction

Environmental sensors play a crucial role in detecting and quantifying physical and chemical parameters in various applications ranging from environmental monitoring and industrial safety to healthcare diagnostics. Among these, thermal sensors [1,2,3,4,5], which measure temperature changes, and gas sensors [6,7,8,9,10,11], which detect specific gas molecules, stand out for their importance in ensuring environmental and human health. Gas-sensing technology is essential for detecting and measuring the presence of various gases in different environments, and it plays a critical role in applications ranging from environmental monitoring to industrial safety and healthcare [12,13]. Accurate gas detection helps prevent accidents, ensures regulatory compliance, and protects public health, making gas sensors indispensable in numerous fields. Gas sensors that can identify specific gases at room temperature are particularly crucial for environmental monitoring, industrial operations, agriculture, military applications, public safety, and medical diagnostics [12,13,14,15]. In environmental monitoring, they track air quality and pollution levels. Industrial applications rely on gas sensors to monitor hazardous gases, ensuring safety in workplaces like chemical plants and refineries. In healthcare, gas sensors enable breath analysis for medical diagnostics and detect toxic gases to ensure the safety of patients and staff in hospitals [15].

Two-dimensional (2D) materials have recently emerged as promising candidates for enhancing gas sensor performance. These materials, which include graphene and transition metal dichalcogenides (TMDCs), are known for their atomic-scale thickness and unique electronic properties. Effective gas sensors must possess high sensitivity and selectivity to accurately distinguish between different gases. They should also have fast response and recovery times, to provide timely measurements. Stability and reproducibility are crucial for ensuring consistent performance over extended periods and under varying conditions. The advent of 2D nanomaterials has sparked a significant revolution in gas sensing. These materials are particularly suited for gas adsorption as a result of their large surface-to-volume ratio, large specific surface area, and highly reactive surfaces. They also hold significant potential for integration with complementary metal–oxide–semiconductor (CMOS) fabrication and exhibit high sensitivity to gases at room temperature. Various 2D materials have shown promise for integration into digital logic field-effect transistors (FETs) and sensing films, due to their wide bandgap [16]. Consequently, for optimized applications, gas sensors must meet two critical criteria: sensitive detection of the presence of a gas and provision of a discernible signal indicating the qualitative or quantitative attributes of the gas [17]. 2D materials are characterized by their one-atom-thick structure, providing a large surface area relative to their volume. They can be classified into various categories, such as graphene oxide, reduced graphene oxide, and transition metal dichalcogenides (TMDCs) [16,18]. These materials exhibit unique properties, such as high electrical conductivity, mechanical strength, and chemical stability. The large surface area of 2D materials allows for significant gas adsorption, improving sensitivity. Their electronic properties can change in response to gas molecules, enabling for precise detection. These features enhance the performance of optically activated gas sensors by increasing their sensitivity and selectivity. In environmental monitoring, optically activated gas sensors using 2D materials can detect pollutants, such as nitrogen oxides (${\mathrm{NO}}_{x}$), sulfur dioxide (${\mathrm{SO}}_{2}$), and volatile organic compounds (VOCs). This capability is critical for assessing air quality and mitigating the effects of pollution. In industrial settings, optically activated gas sensors help detect hazardous gases, such as methane (${\mathrm{CH}}_{4}$), hydrogen sulfide ($H_{2}$S), and ammonia (${\mathrm{NH}}_{3}$), improving safety by providing early warning of gas leaks and preventing accidents. In healthcare, gas sensors are used for breath analysis, offering non-invasive diagnostic tools for diseases like diabetes and lung cancer. They also detect toxic gases, ensuring safe environments in medical facilities and residential areas [14].

**Figure 1:**
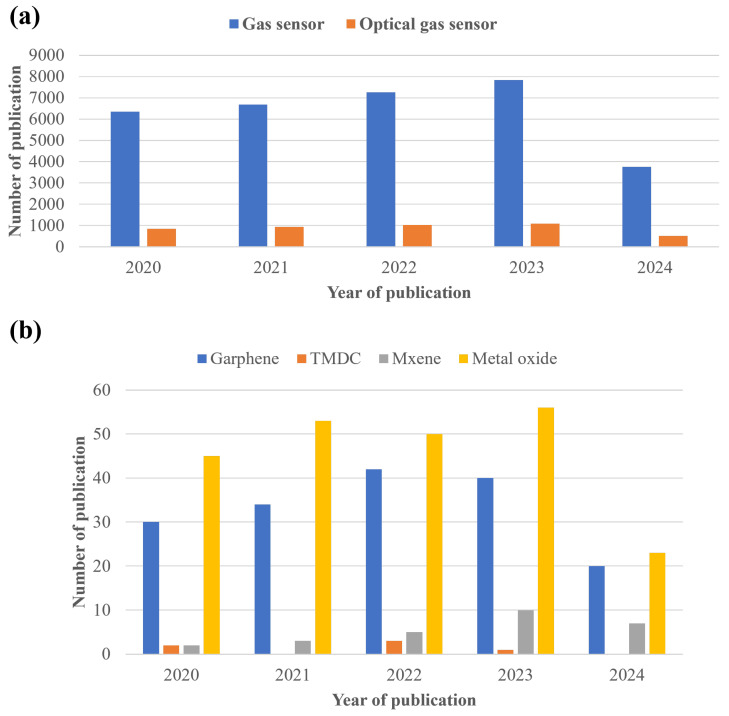
Number of publications from 2020 to 2024 based on Scopus: (**a**) comparison of gas sensor and optically activated gas sensor; (**b**) different 2D-based gas sensors and metal oxides (internet search of the WoS on 3 June 2024).

**Figure 2:**
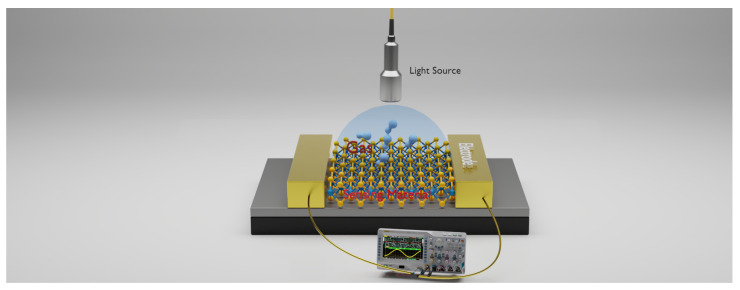
Schematic illustrating a general structure of an optically activated sensor where the light-assisted reaction induces a measurable change that can detect a target gas, usually in the form of a resistance change that can be further processed in an integrated setup, to detect and monitor the gas in applications of interest.

As indicated in Figure 1a, there are a number of publications on traditional gas sensors, indicating a well-established field. However, the comparatively low number of studies on optically activated gas sensors, especially using advanced 2D materials, suggests a significant research gap, as shown in Figure 1b. While the field of gas sensors is well-researched, traditional sensor technology often faces limitations in sensitivity, selectivity, and miniaturization. Consequently, research has shifted to 2D materials like graphene, TMDCs, and Mxene. Given the rapid advancements in nanotechnology and material sciences, focusing on optically activated gas sensors utilizing 2D materials could lead to the development of next-generation sensors with superior performance metrics. The fast response time, selectivity, low limit of detection (LOD), and other important figures of merit (FoMs) offer several advantages for optically activated gas-sensing technology over other types of gas-sensing technologies, making it a preferred choice in various applications. Figure 2 shows the operating principle of optically activated gas sensors. This consists of a light source that activates the sensor surface, enabling interactions between the sensor material and the target gas. The interactions between the sensor and the target gas result in a change in the optical or electrical properties, which is detected and processed by an integrated system connected to the sensor. The measured data can be visualized, analyzed, and used for real-time measurement of the gas concentration. This setup can be integrated into applications requiring precise gas detection and monitoring systems. This review aims to explore the advancements in optically activated gas sensors—focusing on the integration of 2D and organic materials and metal oxide—with a focus on exploring their characteristics and principles of operation. This review is structured as follows: initially, we discuss the gas sensors’ characteristics. Next, we review the varieties of optically assisted gas sensing, including graphene, organic materials, metal oxides, and TMDCs. Finally, we discuss the challenges and the future outlook in the field of optically activated gas sensors.

### Gas Sensor Characteristics

Understanding the key characteristics of gas sensors is essential for selecting the appropriate sensor for specific needs. The main characteristics of gas sensors include response, recovery time, selectivity, response time, stability, and limit of detection. Each of these parameters defines a specific aspect of sensor performance and determines how effectively a gas sensor can detect and quantify the presence of target gases. These characteristics are explained below:

1. Response (sensitivity) refers to the modification in resistance or current when exposed to specific gas molecules. Various techniques can be employed to measure the resulting change in sensor resistance. The response is characterized by the ratio of the sensor’s resistance when in the presence of air $R_{a}$ to its resistance when exposed to target gas molecules $R_{g}$ (i.e., $\frac{R_{a}}{R_{g}}$). This response definition is predominantly applied when the target gas has reducing properties. Conversely, when the gas exhibits oxidative traits, the response is defined as $\frac{R_{g}}{R_{a}}$. In addition to the simple ratio, the relative response can be quantified better, to reflect changes in sensor behavior under varying conditions. For reducing gases, the relative response is calculated as $\left(\frac{R_{a}-R_{g}}{R_{a}}\right)\times 100\%$, which measures the percentage decrease in resistance due to gas exposure. For oxidizing gases, it is determined by $\left(\frac{R_{g}-R_{a}}{R_{a}}\right)\times 100\%$, representing the percentage increase in resistance [8].
2. Recovery time is the duration a gas sensor takes to return to its baseline resistance or current after the removal of the target gas. This parameter indicates how quickly a sensor can be ready for the next detection cycle [19].
3. Selectivity refers to the characteristic that analyzes the capacity of a gas sensor to isolate a particular gas from a mixture of gases [20]. It can be expressed as the ratio of the sensor’s response to the target gas ($S_{target}$) to its response to an interfering gas ($S_{interfering}$) [21]:
  (1) $$S_{el}=\frac{S_{target}}{S_{interfering}}$$
4. Response time is the amount of time taken by a gas sensor when the concentration of a gas reaches a certain value, with respect to the time that it takes to compel the sensor to generate an alarm signal [20].
5. Stability is the characteristic that tells whether the given sensing material can reconcile to its initial state once the detection is over. This includes retaining the response time, recovery time, sensitivity, and selectivity [20]. This can be quantified by measuring the sensor’s response over time and ensuring it remains consistent. This can involve calculating the standard deviation (σ) of the response over time and ensuring it stays within acceptable limits. A common expression for stability is [22]
  (2) $$S_{t}=\frac{\sigma }{meanresponse}\times 100\%$$
6. The limit of detection (LOD) refers to the smallest concentration of a gas that can be reliably detected by the sensor. It represents the lowest amount of analyte that produces a signal distinguishable from the noise or baseline of the sensor system. The LOD is a crucial parameter for evaluating the performance of a gas sensor, as it determines the sensor’s sensitivity and its ability to detect trace amounts of gases. The LOD is typically expressed in units, such as parts per million (ppm), parts per billion (ppb), or even parts per trillion (ppt), depending on the sensitivity of the sensor and the application requirements. A lower LOD indicates a more sensitive sensor, capable of detecting smaller concentrations of the target gas. The LOD can be mathematically represented as [23]
  (3) $$LOD=\frac{3\times RMSnoise}{Sensitivity}$$

## 2. Optically Assisted Gas Sensing

In gas sensing, sensors are designed to measure concentrations for the qualitative and quantitative analyses of gas molecules. The designs can be broadly categorized into various types, including optical, chemi-resistive, field-effect transistor (FET)-based, electrochemical, capacitance-based, and others. Among these different types of gas sensors, optically activated gas sensors have gained significant attention because of their unique advantages, such as their high sensitivity, selectivity, stability, and insensitivity to environmental changes [24]. Unlike chemi-resistive, FET-based, electrochemical, and capacitance-based sensors, which rely on electrical signal changes, optically activated gas sensors detect variations in light properties, such as absorption or refractive index, when gas molecules interact with the sensing element. This method offers a significant advantage in sensitivity, often reaching ppb levels or even lower. Moreover, the advent of scalable, low-power LED sources has paved the way for enhanced gas detection in semiconductor metal oxides through photoactivation. Leveraging photoactive nanomaterials in gas sensors introduces an innovative strategy to improve critical attributes, such as durability, selectivity, and sensitivity, while substantially reducing power consumption. This approach also presents a practical and energy-efficient alternative to conventional thermal-based gas-detection techniques, positioning it as a promising solution for next-generation sensor technologies.

In optical gas sensing, techniques like tunable diode laser absorption spectroscopy (TDLAS) are particularly applicable for their ability to detect trace amounts of gases with high precision. For example, as demonstrated by Minxia Mao et al., TDLAS can target specific absorption lines of gas molecules, allowing it to detect very small concentrations that might go unnoticed by other sensors [25]. Here, when the gas molecules interact with the incident light, they either absorb or emit specific wavelengths. The detector then analyzes these changes in the light properties, to determine the gas concentration [17]. In addition to their remarkable sensitivity, optically activated gas sensors are crucial for accurately identifying specific gases in complex mixtures. This selectivity is achieved by targeting optical properties unique to certain gas molecules, effectively minimizing cross-sensitivity and interference from other gases. For instance, TDLAS and other optical devices can be precisely tuned to specific wavelengths corresponding to the absorption lines of the target gas, enabling them to distinguish the target gas from other components in the mixture. In contrast, as investigated by Ronil J. Rath et al., chemi-resistive and electrochemical sensors often struggle with selectivity, as the changes in electrical resistance or electrochemical reactions they rely on can be triggered by multiple gases, leading to potential false positives. As a result, they may not differentiate between various gases having similar physiochemical properties in uncontrolled conditions [26]. Moreover, the high selectivity of optically activated gas sensors makes them particularly valuable in applications where accurate gas identification is critical, such as in biosensor applications [27].

Optically activated gas sensors are not only sensitive and selective, but also remarkably versatile, with applications spanning environmental monitoring, industrial safety, medical diagnostics, and beyond. In environmental monitoring, these sensors detect trace levels of pollutants, including nitrogen oxides ${\mathrm{NO}}_{x}$ and sulfur dioxide ${\mathrm{SO}}_{2}$, with exceptional precision. These applications highlight the broad utility of optically activated gas sensors and demonstrate their ability to address critical and diverse needs in various industries. Given their noble applications, this review focuses specifically on optically activated gas sensors, exploring their working principles, implications, and potential applications.

The subsequent sections of this Review provide an in-depth and systematic analysis of diverse optically activated gas sensors that encompasses a range of materials and technologies. We begin with a detailed exploration of two-dimensional (2D) materials utilizing graphene, followed by a thorough examination of optically activated gas sensors utilizing organic materials. Next, we delve into metal oxide-based optically activated gas sensors, and, finally, we conclude with a comprehensive discussion of transition metal dichalcogenide (TMDC)-based sensors, ensuring thorough coverage of the field. Integrating 2D nanostructures, such as TMDCs, graphene, and metal oxides, can significantly improve the performance of optically activated gas sensors. These materials boast unique electronic, optical, and surface properties, making them ideal for augmenting the sensitivity and selectivity of gas sensors. Incorporating 2D nanomaterials not only amplifies the functional capabilities of optically activated gas sensors but also enables the development of flexible, miniaturized devices deployable in diverse environments and applications. Compared to traditional bulk materials, 2D nanomaterials offer distinct advantages in gas-sensing applications, particularly when optically activated. Their high surface-to-volume ratio provides more active sites for gas adsorption, directly yielding higher sensitivity and improved detection capabilities.

### 2.1. Optically Activated Gas Sensors Based on Graphene

Traditional detection methods, such as those based on refractive index changes, often suffer from a lack of specificity [28]. Absorption spectroscopy offers a more accurate identification of analytes through their unique absorption spectra. However, commonly used materials like germanium photodetectors are limited in their detection range, particularly for mid-wave infrared (MWIR) photons, due to their higher bandgap [9]. Although III-V photodetectors can detect MWIR radiation, they face significant integration challenges with silicon-based systems [29]. In recent years, two-dimensional (2D) materials, particularly graphene, have emerged as promising candidates for advanced gas sensors. These materials offer remarkable properties, such as high sensitivity, large surface-to-volume ratio, and excellent electrical conductivity, making them ideal for detecting low concentrations of gases. Innovations in graphene-based sensor designs, including integration with silicon and functionalization with various compounds, have led to significant advancements in sensitivity, selectivity, and response times. This section explores the current state of graphene-based gas sensors, highlighting their potential applications and addressing the challenges associated with their development.

**Figure 3:**
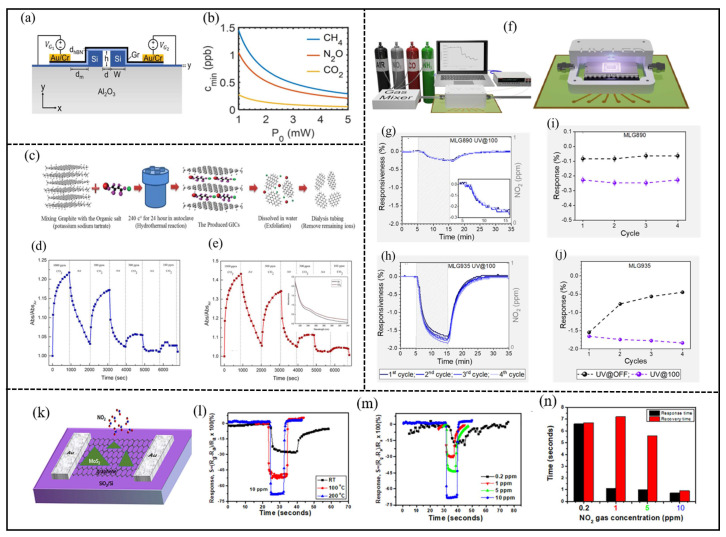
(**a**) Schematic diagram of the on-chip graphene photodetector; (**b**) the graph of minimum detectable concentration ($c_{min}$) versus source power; (**c**) schematic diagram of overall synthesis processes; (**d**) the response–recovery curve of GQD gas sensor at 260 nm; (**e**) the response–recovery curve of GQD gas sensor at 310 nm, with the inset showing the absorption spectra when exposed to air and ${\mathrm{CO}}_{2}$; (**f**) schematic representation of the experimental setup of multilayer graphene (MLG)-based gas sensors; (**g**) real-time responsiveness towards 1 ppm of ${\mathrm{NO}}_{2}$ of MLG890 under UV@100 (**h**) MLG935 under UV@100; (**i**) a comparison of the effect of UV irradiation for MLG890; (**j**) a comparison of the effect of UV irradiation for MLG935; (**k**) schematic of fabricated sensor using ${\mathrm{MoS}}_{2}$/graphene hybrid; (**l**) sensor response to 10 ppm ${\mathrm{NO}}_{2}$ concentrations at different working temperatures; (**m**) sensor response for various ${\mathrm{NO}}_{2}$ concentrations at 200 °C; (**n**) estimated response/recovery time of sensor at 200 °C. Reproduced from Refs. [23,33,34,38].

Two-dimensional materials exhibit high sensitivity to changes in the surrounding environment, rendering them ideal for detecting subtle variations in gas concentrations. The heightened sensitivity enables the detection of low gas concentrations, a critical requirement for applications demanding high sensitivity and low detection limits. Two-dimensional materials, like black phosphorus (BP), offer the potential for integration with silicon via van der Waals forces [30], though BP is unstable in ambient conditions [31]. Graphene is one of the most promising materials for next-generation gas sensing, due to its exceptional properties, including mechanical strength and flexibility, high surface-to-volume ratio, significant conductivity, and low electrical noise. Graphene, a gapless semiconductor, can detect a wide range of electromagnetic radiation and is a promising alternative, though its responsivity in the mid-wave infrared (MWIR) range is generally low compared to telecom wavelengths [32]. Mohammed Alaloul and colleagues proposed an MWIR graphene photodetector integrated into silicon-on-sapphire slot waveguides for on-chip absorption spectroscopy (Figure 3a). The design leverages the presence of the guided mode in the slotted air region, which allows for significant interaction with the analyte gas—an important feature for effective absorption spectroscopy. The device’s performance was evaluated by examining its minimum detectable concentrations for gases such as carbon dioxide (${\mathrm{CO}}_{2}$), methane (${\mathrm{CH}}_{4}$), and nitrous oxide ($N_{2}$O). The results indicated that the photodetector achieved a high responsivity of 0.21 A/W. Figure 3b shows that the proposed design can detect minimum concentrations of 0.14 ppb for ${\mathrm{CO}}_{2}$, 0.52 ppb for $N_{2}$O, and 0.72 ppb for ${\mathrm{CH}}_{4}$ at a source power of 2 mW [33]. Recent research has explored graphene-based optically activated gas sensors, demonstrating their potential for sensitive and versatile detection. Graphene quantum dots (GQDs) have shown promise for room-temperature ${\mathrm{CO}}_{2}$ detection, exhibiting significant sensitivity and partial reversibility [34]. Raeyani et al. developed an optically activated gas sensor, using GQDs that operated at room temperature for detecting carbon dioxide (${\mathrm{CO}}_{2}$) [34]. The schematic diagram of overall synthesis processes is shown in Figure 3c. The sensor showed a significant change in optical absorption when exposed to ${\mathrm{CO}}_{2}$ at an absorption wavelength of 260 nm, with a partially reversible response (Figure 3d). From Figure 3d, it is clear that relative optical absorbance increased when the sensor was exposed to ${\mathrm{CO}}_{2}$ and recovered near the baseline when it was exposed to air. This characteristic suggests that the sensor could be effectively used for ${\mathrm{CO}}_{2}$ detection. The sensor exhibited an average response time of 106 s and an average recovery time of 150 s, both of which were faster than those previously reported for graphene-based optically activated gas sensors [11]. For a ${\mathrm{CO}}_{2}$ concentration of 1000 ppm, the sensor achieved a response of approximately 50% at an absorption wavelength of 310 nm (Figure 3e). The sensor also responded to lower ${\mathrm{CO}}_{2}$ concentrations, highlighting its sensitivity. At shorter wavelengths, the level of change in optical absorption intensity during air and gas exposure was lower than longer wavelengths (inset of Figure 3e). Consequently, the sensing response in shorter wavelengths was lower than longer wavelengths. The calculated response in the wavelength of 310 nm for the concentration of 500, 300, and 100 ppm was about 40%, 8%, and 4%, respectively [11]. Theoretical designs have proposed integrating graphene with silicon core fibers for mid-infrared gas sensing, offering improved sensitivity and low detection limits for gases like nitrogen dioxide [35]. Graphene and its derivatives have been utilized in various optical sensing applications, including fluorescence-based detection, surface-enhanced Raman scattering, and optical fiber biosensors, due to their unique optical properties and biocompatibility [36]. Additionally, tapered optical fibers coated with graphene oxide have demonstrated gas-detection abilities across a wide optical range, with notable sensitivity in the visible spectrum for gases like nitrogen, hydrogen, and propane–butane mixtures [37]. Gas sensors employing multi-layered graphene (MLG) can effectively monitor ${\mathrm{NO}}_{2}$ levels at room temperature (RT). However, detecting concentrations as low as parts per million (ppm) and below presents challenges, due to incomplete recovery and a lack of sensor reproducibility after exposure. To address these issues, Alvaro Pena and his colleagues utilized UV light [23]. The author investigated the impact of UV irradiation on the operation of two devices (MLG890 and MLG935). In Test2, the devices underwent four consecutive cycles of exposure to 1 ppm of ${\mathrm{NO}}_{2}$ under UV light at 100 mW/cm^2^ (UV@100), mirroring the conditions of Test1, which involved the same exposure without UV irradiation (UV@OFF). The results, shown in Figure 3g,h, revealed that the devices’ responsiveness under UV irradiation reached a steady state during the exposure phase, differing from the behavior observed in Test1. Both devices exhibited a significantly higher response under UV irradiation compared to UV@OFF. For the MLG890 device, the average response during exposure increased dramatically from 0.07 ± 0.02% (UV@OFF) to 0.24 ± 0.02% (UV@100), representing a 340% increase. Similarly, for the MLG935 device, the average response rose by 290%, from 0.58 ± 0.15% to 1.70 ± 0.05% under UV irradiation. No significant decrease in response was noted after each cycle, and a full recovery to initial conditions was observed during the purge phase under UV@100, typically within the first 10 min. Additionally, there was no evidence of hysteresis after each cycle of exposure to 1 ppm of ${\mathrm{NO}}_{2}$, suggesting that UV irradiation greatly enhances the reproducibility and reliability of the devices’ responses. These effects, including improved responsiveness and reproducibility, are illustrated in Figure 3i,j, which compare the devices’ responses with UV irradiation (violet dots) and without (black dots) [23]. In summary, the results obtained show that when their proposed MLG devices were exposed to ${\mathrm{NO}}_{2}$ under UV light compared to dark conditions, the sensor response was enhanced by 290% to 550% and the detection limit was reduced to 30 ppb. Hong et al. conducted large-scale thermal chemical vapor deposition (CVD) to synthesize molybdenum disulfide (${\mathrm{MoS}}_{2}$) on graphene, creating a hybrid material used to construct a resistive ${\mathrm{NO}}_{2}$ gas sensor, illustrated in Figure 3k [38]. This sensor demonstrated remarkable sensitivity and selectivity, with a low detection limit of 0.2 ppm. It also exhibited broad detection and operational temperature ranges, from 0.2 to 100 ppm and 25 to 200 °C, respectively. The outstanding performance of this sensor was attributed to the synergistic effects between ${\mathrm{MoS}}_{2}$ and graphene, where the high active surface area of ${\mathrm{MoS}}_{2}$ exposed a large fraction of edge sites, thereby enhancing activity. Figure 3l,m depict the typical response of the sensor at various temperatures and gas concentrations. In the presence of ${\mathrm{NO}}_{2}$, the resistance of the ${\mathrm{MoS}}_{2}$/graphene hybrid sensor decreased rapidly and recovered when ${\mathrm{NO}}_{2}$ was purged from the chamber with air. The sensor’s response to a single cycle of 10 ppm ${\mathrm{NO}}_{2}$ was distinctly influenced by temperature variations, as shown in Figure 3l. Overall, the ${\mathrm{NO}}_{2}$ sensor exhibited a fast response and recovery time, typically within a few seconds, as demonstrated in Figure 3m,n. Zhang et al. combined the exceptional gas-sensing properties of tin(IV) oxide (${\mathrm{SnO}}_{2}$) and zinc oxide (ZnO) with the superior electrical properties of reduced graphene oxide (rGO), to prepare palladium (Pd)-doped rGO/ZnO-${\mathrm{SnO}}_{2}$ nanocomposites, using a hydrothermal method [39]. The study confirmed the successful synthesis of Pd-doped ZnO-${\mathrm{SnO}}_{2}$ composites, which were uniformly coated on the surface of the rGO. The hydrogen-gas-sensing performance of the sensor was evaluated, revealing that the Pd-doped rGO/ZnO-${\mathrm{SnO}}_{2}$ sensor modified with 3 wt% rGO exhibited a significantly improved hydrogen ($H_{2}$) sensing response, detecting $H_{2}$ concentrations ranging from 9.4 to 100 ppm at 380 °C compared to the pure Pd-doped ZnO-${\mathrm{SnO}}_{2}$ sensor. Additionally, the sensor demonstrated extremely fast response and recovery times (4 s and 8 s, respectively, for 100 ppm $H_{2}$ at 380 °C) and a remarkably low detection limit of 50 ppb. The sensor also showed excellent repeatability and recovery characteristics. The enhanced sensing performance of the Pd-doped rGO/ZnO-${\mathrm{SnO}}_{2}$ sensor was primarily attributed to the heterostructure formed by rGO, ZnO, and ${\mathrm{SnO}}_{2}$, the outstanding electrical and physical properties of rGO, and the synergistic interaction between rGO and Pd.

While graphene-based gas sensors have already demonstrated high sensitivity, achieving selectivity remains a key challenge. Selectivity refers to a gas sensor’s ability to respond more strongly to a specific target analyte than to multiple interfering gases. This means the sensor’s response to the target gas should be greater than its response to other gases. In graphene chemi-resistors, the sensing mechanism involves changes in graphene’s conductivity when exposed to various gases. In recent years, functionalizing the graphene surface has been proposed as a solution to this selectivity issue. This approach relies on the different interaction strengths between analytes and functional species, which can preferentially bind to the target gas. Various adsorbates, including metal oxides, metal nanoparticles, polymers, and organometallic molecules, have been successfully used to enhance sensitivity, recovery time, and, especially, selectivity. Graphene can be functionalized both covalently and noncovalently. Covalent functionalization involves forming chemical bonds between graphene and target molecules through reactions such as nucleophilic addition, cyclo-addition, condensation, and electrophilic reactions. Noncovalent functionalization attaches functional groups to the graphene surface, based on electrostatic interactions, such as van der Waals forces or π-π interactions. Aromatic molecules with planar structures are ideal candidates for noncovalent functionalization, due to their ability to anchor to the graphene surface through π-π interactions [6]. A variety of graphene hybrids have been studied, to improve the performance of bare graphene gas sensors. These hybrids include organic molecules like porphyrins and phthalocyanines, as well as conductive polymers like polypyrrole. Porphyrins are macrocyclic compounds with an 18-π-electron conjugated ring system that can be transformed into metalloporphyrins (MPs) by substituting two hydrogen atoms with a transition metal atom at the core. MPs have strong catalytic activity, making them effective functional materials that enhance the chemical response of graphene. Phthalocyanines are 2D, 18-π-electron materials with high electronic delocalization. Metallophthalocyanines (MPcs) are formed by substituting hydrogen atoms with a transition metal. Their thermal and chemical stability, along with a highly tunable structure, make MPcs excellent candidates for gas sensing. Polypyrrole (PPy) is a conjugated polymer that has emerged as a promising material for sensing applications, due to its low cost, easy synthesis, moderate conductivity, and ability to detect a wide range of volatile compounds. Additionally, PPy can operate at room temperature, which is a significant advantage for practical applications [6]. Mackin et al. reported a sensor system capable of rapidly and conveniently measuring hundreds of functionalized graphene sensors. This system utilized a novel array architecture that required only one sensor per pixel and eliminates the need for a selector transistor. The system specifically evaluated Co(tpfpp)${\mathrm{ClO}}_{4}$-functionalized graphene sensors for ammonia detection, building on previous research [40]. The Co(tpfpp)${\mathrm{ClO}}_{4}$-treated graphene sensors demonstrated a fourfold increase in ammonia sensitivity compared to pristine graphene sensors. Additionally, these sensors exhibited excellent selectivity against interfering substances, such as water and common organic solvents. Monitoring a large sensor array with 160 pixels provided valuable insights into performance variations and reproducibility, which are critical for developing practical sensor systems. In another study, Co-porphyrin functionalized graphene sensors were fabricated to achieve selective ammonia (${\mathrm{NH}}_{3}$) sensing via the selective metal–ligand bond between cobalt (Co) and ${\mathrm{NH}}_{3}$ [41]. The selectivity of these sensors was tested with mixtures of hydrogen ($H_{2}$) and ammonia (${\mathrm{NH}}_{3}$). The sensors successfully detected sub-ppm levels of ${\mathrm{NH}}_{3}$ (1 ppm) without responding to high concentrations of $H_{2}$ (400 ppm). Additionally, the effect of humidity on the Co-porphyrin functionalized graphene ${\mathrm{NH}}_{3}$ sensor was investigated, demonstrating reliable detection of low concentrations of ${\mathrm{NH}}_{3}$ even under rapidly changing humidity conditions. These functionalized sensors showed a sixfold greater response to ammonia compared to non-functionalized graphene. The key sensing mechanism was attributed to charge transfer to graphene, due to the electronic structure changes in Co-porphyrin-graphene complexes upon ${\mathrm{NH}}_{3}$ adsorption on Co-porphyrin. Zhou et al. proposed ammonia gas sensors based on reduced graphene oxide (rGO) functionalized with copper phthalocyanines (3-CuPc), specifically 3-CuPc-1,8,15,22-tetra-iso-pentyloxyphthalocyanine copper. The hybrids were prepared by reducing graphene oxide (GO) with hydrazine in the presence of 3-CuPc. Characterization using UV-vis and X-ray photoelectron spectroscopy (XPS) confirmed the noncovalent attachment of 3-CuPc molecules to the rGO surface via π-π stacking interactions. The sensor response to various ${\mathrm{NH}}_{3}$ concentrations (400 ppb to 3200 ppm) was compared with pristine rGO. The 3-CuPc/rGO sensors exhibited a response to ${\mathrm{NH}}_{3}$ that was 15 times higher than to other gases, such as ${\mathrm{CO}}_{2}$, ${\mathrm{CH}}_{4}$, $H_{2}$, and CO. This enhanced performance was attributed to charge transfer between rGO, 3-CuPc, and ${\mathrm{NH}}_{3}$, with electrons transferring from ${\mathrm{NH}}_{3}$ to the phthalocyanine molecules, which served as active sites for ${\mathrm{NH}}_{3}$ adsorption. The efficient charge transfer between 3-CuPc and rGO contributed to the high sensitivity of the sensors [42]. Guo et al. investigated the gas-sensing performance of four rGO-based nanohybrids with cobalt phthalocyanines (CoPc) containing different phenoxyl substituents (cpoCoPc, poCoPc, cmpoCoPc, and mpoCoPc). These hybrids, along with rGO and phthalocyanine films, were tested for 18 different gases, including ${\mathrm{NH}}_{3}$. The cpoCoPc/rGO hybrid showed the highest response, with 42.4% for 100 ppm ${\mathrm{NH}}_{3}$, a reversible recovery time of 120 s, and a low detection limit of 3.7 ppb. The study highlighted the significant selectivity to ${\mathrm{NH}}_{3}$ for all hybrids compared to other gases [43]. The effect of different substituted phthalocyanines on the ${\mathrm{NH}}_{3}$-sensing performance of rGO/Pcs hybrids was also evaluated. Three sensors were fabricated, using cobalt amino phthalocyanines (ABOCoPc, ACoPc), substituent-free cobalt phthalocyanine (FCoPc), and rGO. The CoPc/rGO sensors demonstrated superior response and recovery times compared to the rGO sensors alone. Furthermore, the hybrid sensors showed selectivity towards ${\mathrm{NH}}_{3}$ over 16 other tested gaseous compounds (VOCs and ${\mathrm{NO}}_{x}$). The performance was linked to the amino groups in the ACoPc, which acted as strong electron donors, facilitating electron transfer and enhancing sensor sensitivity [44]. Li et al. described the fabrication of hybrid sensors combining metallophthalocyanines (MPc) and reduced graphene oxide (rGO) with varying metallic centers (M = Ni, Cu, Pb). Among these, NiPc/rGO exhibited the fastest response time, while CuPc/rGO had the fastest recovery time. All MPc/rGO hybrids fully recovered to their original resistance, unlike the rGO sensors. Yu et al. reported that MPc/rGO hybrids could detect ${\mathrm{NH}}_{3}$ at concentrations as low as 400 ppb. These sensors demonstrated weak responses to ${\mathrm{CO}}_{2}$, ${\mathrm{CH}}_{4}$, $H_{2}$, and CO, confirming their selectivity towards ${\mathrm{NH}}_{3}$ [45]. Another study explored hybrids based on rGO and tetrakis(4-tert-butylphenoxy)phthalocyanine (TBPOMPc) with different metallic centers (M = Cu, Ni, Pb). X-ray photo-electron spectroscopy (XPS) confirmed the noncovalent functionalization of the rGO. These sensors, tested with ${\mathrm{NH}}_{3}$ concentrations ranging from 0.3 to 3200 ppm at room temperature, showed an increase in resistance with higher ${\mathrm{NH}}_{3}$ concentrations and fully recovered to their original resistance, unlike pure rGO sensors. The TBPOMPc/rGO hybrids were selective towards ${\mathrm{NH}}_{3}$ over CO, ${\mathrm{NO}}_{2}$, and $H_{2}$ [46]. Kumar et al. developed a selective room-temperature chemi-resistor by functionalizing rGO with CuPc nanoflowers, achieving a ppb detection limit for ${\mathrm{Cl}}_{2}$. The functionalization involved mixing CuPc powder with GO, followed by reduction with hydrazine. Fourier-transform infrared (FTIR) measurements indicated noncovalent π-π interactions between CuPc and the rGO surface. The hybrid material exhibited high selectivity for ${\mathrm{Cl}}_{2}$ compared to other gases, like ${\mathrm{NO}}_{2}$, NO, and ${\mathrm{NH}}_{3}$. At a low concentration of 3000 ppb ${\mathrm{Cl}}_{2}$, the sensor showed a significant response, with a response/recovery time of 190 s/545 s [47].

In summary, graphene-based gas sensors represent a significant advancement in the field of gas detection, offering unparalleled sensitivity, selectivity, and versatility. The unique properties of graphene, such as its large surface area, excellent electrical conductivity, and flexibility, make it an ideal material for detecting a wide range of gases at low concentrations. Despite the challenges associated with achieving selectivity and stability, recent innovations, including the use of functionalization techniques and integration with other materials, have shown promising results. These advancements have enabled the development of sensors capable of detecting specific gases with high accuracy and reliability. The ongoing research into graphene hybrids and novel device architectures continues to push the boundaries of gas sensor performance, paving the way for practical applications in environmental monitoring, industrial safety, and medical diagnostics. As the field progresses, further improvements in the design and fabrication of graphene-based sensors are expected, addressing existing limitations and expanding their range of applications. Ultimately, the integration of graphene-based gas sensors into commercial devices has great potential to enhance our ability to monitor and respond to changes in the environment, ensuring a safer and more sustainable future.

### 2.2. Optically Activated Gas Sensors Based on Organic Materials

Despite significant advancements in gas-sensing technologies, there is a noticeable scarcity of comprehensive studies and literature focusing specifically on optically activated gas sensors that utilize organic materials. Optically activated gas sensors generally function based on variations in absorbance and luminescence caused by gas analytes interacting with a sensitive material. Despite the potential application of organic materials as sensors, they face challenges, such as poor stability, low surface area, and limited sensitivity at room temperature [48]. However, researchers have reduced some of these issues by developing hybrid organic–inorganic sensors that offer high detection sensitivity alongside improved durability. Henceforth, pure organic materials may not be optimal for optically activated gas sensors, since the ongoing advancement in materials and sensor technology is continuously improving their performance [48].

**Figure 4:**
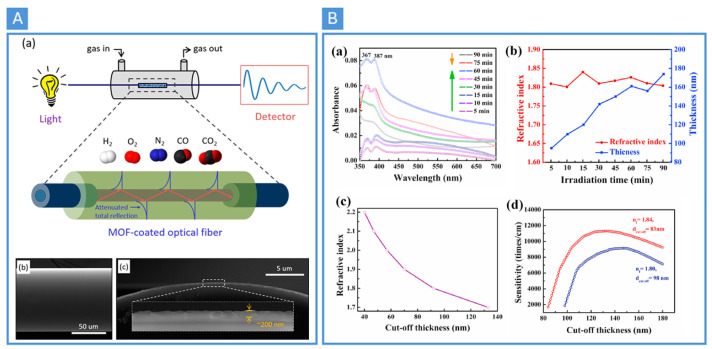
(**A**) (**a**) Optical-fiber sensor combined with MOF thin film and gas-sensing system schematic diagram; (**b**) top and (**c**) 200 nm ZIF-8-coated optical-fiber cross-sectional FE-SEM pictures showing the continuous and homogeneous thin layer [50]. (**B**) (**a**) Absorbance and (**b**) variations in the refractive index and thickness of PR-MOF-1 membrane with varying growth periods; (**c**) hypothesized connection between cut-off thickness and refractive index; and (**d**) thickness and sensitivity of the PR-MOF-1 membrane [53].

The phrase metal–organic frameworks (MOFs) was first used by scientists in 1995, to describe porous coordination polymers made of metal ions and organic ligands. MOFs are characterized by their large specific surface area, high porosity, multiple coordination sites, and versatile, tunable structures, enabling a wide range of functionalities [49]. Therefore, MOFs are extensively utilized in gas-sensing applications, due to their exceptional properties. In 2018, Ki-Joong Kim and his team developed MOF thin-film-coated optical fiber sensors (see Figure 4A) to show a particular sensitivity to carbon dioxide (${\mathrm{CO}}_{2}$) compared to other gases like hydrogen ($H_{2}$), oxygen ($O_{2}$), nitrogen ($N_{2}$), and carbon monoxide (CO), due to the changes in the refractive index of the MOF film upon gas adsorption [50]. The fibers were first cleaned and etched, followed by the deposition of MOF films. The authors chose zeolitic imidazole framework material (ZIF-8), a type of MOF known for its stability and selectivity, as the coating material [50]. In the same year, Jiri Hromadka et al. used a long-period grating (LPG) on optical fiber that had been altered by a thin layer of zeolitic imidazole framework material (ZIF-8), which is part of the metal–organic framework family, to detect organic vapors [51]. After two years, in 2020, Rongtao and his team developed coatings that react to gas for both single and multi-mode optical fibers, using polymer/nanocrystalline metal–organic framework composites, specifically designed for methane (${\mathrm{CH}}_{4}$) sensing in natural-gas infrastructures. The use of silicone polymers based on polydimethylsiloxanes (PDMS) combined with ZIF-8 resulted in optimized optical and mechanical properties, enhancing the solubility and permeability of ${\mathrm{CH}}_{4}$ in the resulting film [52]. In 2021, Nizamadin et al. developed a photoresponsive metal–organic framework (MOF) membrane, using UV light irradiation. Upon 60 min of UV exposure, the morphology of the photo-responsive metal–organic framework (PR-MOF-1) changed into a densified ladder-layered structure from a porous honeycomb structure (see Figure 4B) [53]. As measured with an asymmetric planar optical waveguide gas sensor, the as-grown film was optically transparent and showed a higher sensing response to ethylenediamine (EDA) gas, even in the presence of interfering substances like ammonia and dimethylamine, as well as benzene, xylene, toluene, and styrene gases [53].

Organic conducting polymers (CPs), including polypyrrole (PPy), polythiophene (PTh), polyaniline (PANI), polyacetylene (PA), and poly (3,4-ethylenedioxythiophene) (PEDOT) are candidate materials for fabricating gas sensors [54]. The improved characteristics of CP sensors’ active layers include their high sensitivity, ease of synthesis, easy fabrication, due to their strong mechanical properties, re-usability, lower detection threshold value, and room-temperature operation [54]. In order to monitor ${\mathrm{NH}}_{3}$, ethanol, and acetone gases at room temperature, Muthusamy and colleagues developed gas sensors using a fiber optic coated with PPy/Prussian blue (PPy-PB) and PPy nanocomposite [55]. Another class of optical sensors, known as surface plasmon resonance (SPR) optical sensors, was used to activate surface plasmon-based optical sensors for light-based chemical sensing [55]. The sensing device, based on the SPR optical sensor, uses a thin-film refractometer to determine the variation in the refractive index of a metal film reinforced by plasmons. It is triggered by monochromatic illumination, which causes a dielectric material’s refractive index to vary and causes a variation in the external plasmon propagation constant [56]. The parameters of the light waves connected to the surface plasmon are adjusted by the radiation propagation constant. The minimum in the reflectance curve shifts after interacting with analytes, indicating the analyte residency [57].

**Figure 5:**
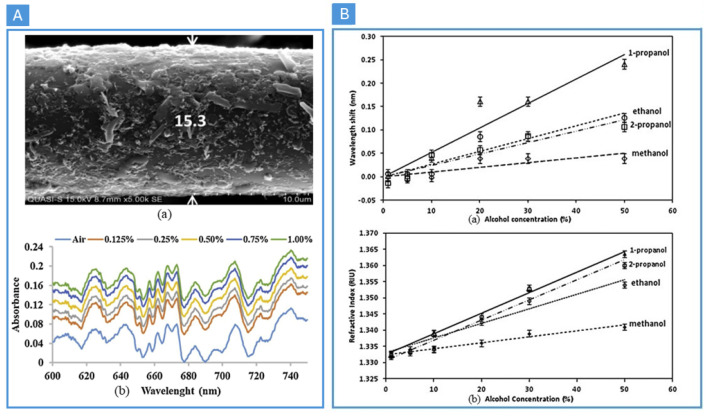
(**A**) (**a**) SEM images of PANI/GNF nanocomposite on the SMF; and (**b**) absorbance spectra of the etched–tapered SMF sensor coated with PANI/GNF nanocomposite towards ${\mathrm{NH}}_{3}$ in the visible wavelengths range [58]. (**B**) (**a**) Wavelength shift for different types of alcohols; (**b**) output response of the sensor to the refractive indices of different alcohols [59].

A high-surface-area PANI/graphite nanofiber (GNF) nanocomposite was recently coated on an etched–tapered single-mode fiber (SMF) by Mohammed et al., to produce an optical sensor for detecting ${\mathrm{NH}}_{3}$ gas at room temperature in the visible wavelength region (see Figure 5A) [58]. When contrasted with pure PANI-coated SMF, the sensor demonstrated good reaction time, sensitivity, and repeatability for ${\mathrm{NH}}_{3}$. Furthermore, PANI doped with dioctyl sodium sulfosuccinate was coated onto a microfiber resonator, to construct an optical microfiber sensor for alcohol detection [59]. Because the PANI fiber’s average band gap (lower energy) and dihedral angle increased in reaction to different alcohols at varying concentrations, the sensor’s return spectrum displayed a red shift in wavelength (see Figure 5B) [59]. In 2019, Wu and his team discovered that the refractive index of an organic polymer (OP) changes with varying concentrations of acid gases that interact with the polymer. To exploit this phenomenon, they placed the OP film inside the Fabry–Perot (F–P) cavity and analyzed the spectral properties of the cavity’s output, to detect hydrogen sulfide gas [60]. Their practical outcome demonstrated that the refractive index of the OP decreased as the gas amount increased. At low concentrations, they achieved a resolution of 3.0455–$4.7777\times 10^{-3}$ m/%VOL, a phase sensitivity of $1.94\times 10^{2}$ rad/%VOL, and a concentration of $5.15\times 10^{-3}$ ppm [60]. This experiment validated the feasibility of using organic polymers for gas sensing. Overall, this approach is suitable for accurate measurement of gases such as ${\mathrm{CO}}_{2}$ and hydrogen sulfide in the hydrocarbon industry [60].

According to past studies, researchers have developed hybrid organic–inorganic sensors that offer high detection sensitivity and improved durability, in spite of obstacles like low surface area, poor stability, and limited sensitivity at room temperature. Due to their remarkable qualities, which include high porosity, a large specific surface area, and adaptable tunable structures, metal–organic frameworks have become highly promising candidates and are broadly used in gas-sensing applications. Numerous studies have shown how these materials can be used to detect various gases, exhibiting high sensitivity and quick response times.

### 2.3. Optically Activated Gas Sensors Based on Metal Oxides

Metal oxides (MOs), which consist of metallic cations and oxygen anions, can be represented as MO, $M_{x}$O, ${\mathrm{MO}}_{x}$, and $M_{x}$$O_{y}$, where *M* denotes metals, *O* denotes oxygen, and *x* and *y* indicate oxidation states [61]. These metal oxide semiconductors (MOSs) have recently garnered significant attention, due to their high sensitivity to various gases, low cost, and easy fabrication processes [62,63]. Since Seiyama’s initial demonstration of gas sensing based on metal oxides [64] extensive efforts have been dedicated to studying the sensing performance of metal oxide-based sensors [65,66,67].

The advent of scalable, low-power LED sources has spurred interest in leveraging photoactivation to enhance gas-detection abilities in semiconductor metal oxides. Employing photoactive nanomaterials in gas sensors represents an innovative approach to improving essential properties, such as durability, selectivity, and sensitivity, while also reducing power consumption. These photoactive sensors also present a feasible alternative to conventional thermal heating in gas-detection processes. Wide-bandgap semiconductors, such as ${\mathrm{SnO}}_{2}$, ${\mathrm{TiO}}_{2}$, and ZnO, typically require high operating temperatures to optimize gas detection; however, under UV irradiation, it is possible to lower these temperatures. For instance, Zeng et al. [68] developed hedgehog-like ${\mathrm{SnO}}_{2}$ nanostructures assembled into nanocones, using a hydrothermal method, and they explored their ability to detect hydrogen at a temperature as low as 50 °C. Their findings revealed that UV irradiation significantly enhances the sensor response at this reduced temperature. Additionally, ${\mathrm{SnO}}_{2}$ thin films prepared by RF sputtering showed resilience and selective adsorption under relatively high humidity operation when used to detect $O_{3}$ of different concentrations under 370 nm illumination [69].

Recent developments in light-activated gas sensors have highlighted the potential for enhancing the performance of ${\mathrm{TiO}}_{2}$-based gas sensors. When exposed to light, the ${\mathrm{TiO}}_{2}$ conductivity increases as electrons are excited from the valence to the conductive band, effectively facilitating the redox interactions between the ${\mathrm{TiO}}_{2}$ and the target gas. This mechanism substantially boosts the sensor’s effectiveness in gas detection by promoting more efficient chemical reactions under UV irradiation [70,71,72,73].

In addition to the aforementioned oxides, ZnO is sought after for room-temperature photo-activated sensors, due to its transparency and complete biocompatibility, and to its distinct optical and catalytic properties [74]. ZnO has been employed for sensing under UV illumination in different structures, including nanoparticles [75], thin and thick films [76], nanorods [77,78], and microspheres [79]. ${\mathrm{In}}_{2}$$O_{3}$, an n-type semiconductor with enhanced electrical conductivity in its non-stoichiometric form has been widely investigated for its gas-sensing properties under UV illumination. In a study by Wagner et al., the enhanced ${\mathrm{NO}}_{2}$-sensing abilities of ordered mesoporous ${\mathrm{In}}_{2}$$O_{3}$ were attributed to photoreduction affecting oxygen vacancy states [80]. Building on this understanding, Ilin et al. prepared nanocrystalline indium oxide films via sol–gel, with grain sizes ranging from 7–40 nm, which exhibited increased ${\mathrm{NO}}_{2}$ sensitivity under UV light. Notably, smaller grains were more responsive, whereas medium grain sizes provided the highest response in the absence of UV light, demonstrating that pulsed illumination can lead to energy-efficient ${\mathrm{NO}}_{2}$ detection [81]. Further advancements were demonstrated by Gonzalez et al., who reported an 80-fold increase in ${\mathrm{NO}}_{2}$ sensitivity using indium oxide nano-octahedra sensors. These sensors utilized mild heating alongside pulsed UV light, achieving significant power savings [82]. Building on the morphological influences on sensor performance, Trocino et al. highlighted that ${\mathrm{In}}_{2}$$O_{3}$ nanostructures created via sol–gel and electrospinning exhibit superior ${\mathrm{NO}}_{2}$ sensing at room temperature, especially under pulsed UV light, underscoring the critical role of morphology in sensor effectiveness [83]. Ma et al. synthesized walnut-shaped ${\mathrm{In}}_{2}$$O_{3}$ nanostructures on interdigitated electrode substrates, using a hydrothermal method followed by thermal treatment. These structures were utilized in a photoelectric gas sensor, achieving an ultra-high sensitivity of 219 to 50 ppm ${\mathrm{NO}}_{2}$ under UV irradiation, with notable stability and selectivity [84]. Similarly, using a hydrothermal method, dense mesoporous ${\mathrm{In}}_{2}$$O_{3}$ nanorod arrays were grown on a porous ceramic substrate, which detected ppb-level ${\mathrm{NO}}_{2}$ with high sensitivity and speed, enhanced by UV light during recovery, providing a cost-effective approach to ${\mathrm{NO}}_{2}$ sensor fabrication [85]. Additionally, the unique properties of porous ${\mathrm{In}}_{2}$$O_{3}$ nanoparticles, such as their large surface areas and pore volumes, have been leveraged in $H_{2}$S gas detection. These nanoparticles facilitate the generation of active oxygen species, enhancing the sensor’s ability to detect $H_{2}$S with a remarkably low limit of 1 ppb and respond strongly to 365 nm UV light, demonstrating excellent reversibility, selectivity, and stability in ambient conditions [86].

On the other hand, recent advancements in ${\mathrm{WO}}_{3}$-based gas sensors have shown significant improvements in sensitivity and selectivity under UV-light irradiation and variable temperatures, as evidenced by fluctuation-enhanced gas sensing and DC resistance measurements [87,88]. These studies have demonstrated that UV irradiation can notably enhance gas-detection performance in environments containing synthetic air, ethanol, and nitrogen dioxide, with notable energy efficiency and the ability to discriminate between different gases. Specifically, pulsed UV light activation has been found to effectively facilitate room temperature gas sensing in ${\mathrm{WO}}_{3-x}$ nanoneedles when exposed to ${\mathrm{NO}}_{2}$, significantly reducing power consumption [89]. Further developments include the use of porous ${\mathrm{WO}}_{3}$ nanofibers synthesized via electrospinning and annealing, which exhibit high acetone sensitivity (1.8–12.5 ppm) with a peak response of 1.79 μA under UV light and variable voltage settings. These fibers are capable of detecting ${\mathrm{NO}}_{2}$ concentrations as low as 700 ppb, with rapid response/recovery times of 33/42 s and excellent repeatability [90]. Additionally, ${\mathrm{WO}}_{3-x}$ nanowires modified with (3-aminopropyl)triethoxysilane (APTES) have shown a UV light-activated response to ${\mathrm{NO}}_{2}$ and ethanol, with sensitivity nearly eight times higher than that of unmodified nanowires. Enhancements were further amplified—approximately fourfold—in APTES-modified ${\mathrm{CeO}}_{2}$–${\mathrm{WO}}_{3-x}$ structures, attributed to the reactive amino groups of the APTES enhancing electron transfer to the metal oxide’s conduction band under UV light [91]. Moreover, improvements in formaldehyde (${\mathrm{CH}}_{2}$O) detection have been achieved using tungsten trioxide nanowires deposited on alumina substrates with interdigitated platinum electrodes via aerosol-assisted chemical vapor deposition (AACVD). These sensors, whether pristine or decorated with various metal nanoparticles (Au, Pt, Au/Pt, Ni, Fe), showed enhanced ${\mathrm{CH}}_{2}$O detection under both no illumination and UV illumination at 394 nm. Although UV irradiation quickened C $H_{2}$O desorption times and reduced baseline shifts, indicating that UV light aids in the adsorption–desorption processes, the combination of metal nanoparticle decoration with UV exposure did not produce a synergistic enhancement in detection capabilities [92].

**Figure 6:**
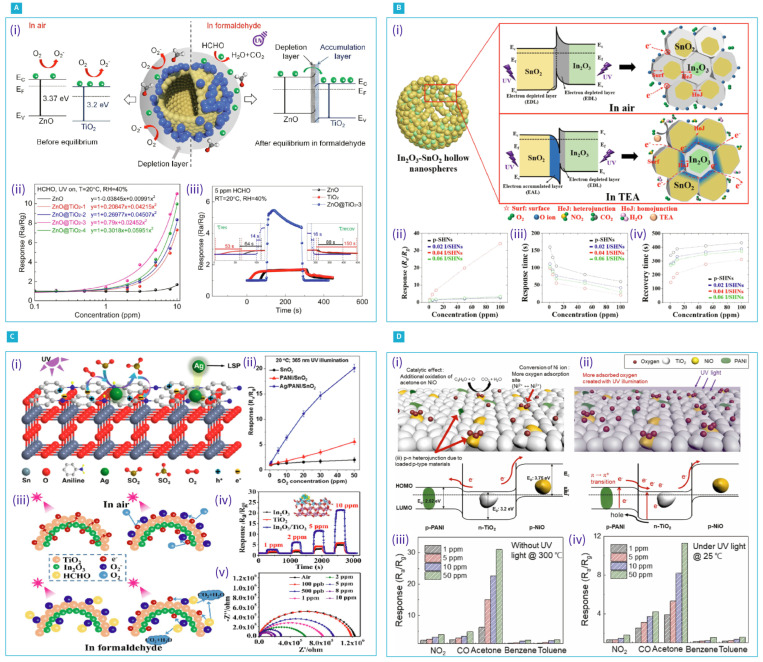
Gas sensors based on MOs heterostructures under UV light activation: (**A**) (**i**) hollow nanospheres ZnO/${\mathrm{TiO}}_{2}$ heterostructure band diagram; (**ii**) response of nanospheres ZnO/${\mathrm{TiO}}_{2}$ heterojunction sensor to varoius concentrations of ${\mathrm{CH}}_{2}$O; (**iii**) time response of pure ZnO, ${\mathrm{TiO}}_{2}$, and ZnO/${\mathrm{TiO}}_{2}$ to 5 ppm ${\mathrm{CH}}_{2}$O at room temperature under 365 nm LED irradiation (“Reprinted from [101], Copyright (2023), with permission from Elsevier”). (**B**) (**i**) Schematic and energy band diagrams of ${\mathrm{In}}_{2}$$O_{3}$/${\mathrm{SnO}}_{2}$ HNSs sensor in air (top) or TEA gas (bottom); (**ii**) sensor responses; (**iii**) response time; and (**iv**) recovery period of synthesized HNs sensors (“pure ${\mathrm{SnO}}_{2}$ HNs (p-SHNs) and 0.02 mol (0.02 I/SHNs), 0.04 mol (0.04 I/SHNs), and 0.06 mol (0.06 I/SHNs) of ${\mathrm{In}}_{2}$$O_{3}$ with ${\mathrm{SnO}}_{2}$ HNs”) at different TEA concentrations under 365 nm UV illumination, respectively (“Reprinted from [102], Copyright (2023), with permission from Elsevier”). (**C**) (**i**) Schematic illustration of the ${\mathrm{SO}}_{2}$ detection mechanism in the 365 nm UV-activated Ag-PANI/${\mathrm{SnO}}_{2}$ sensor; (**ii**) gas-sensing performance of ${\mathrm{SnO}}_{2}$, PANI/${\mathrm{SnO}}_{2}$, and Ag-PANI/${\mathrm{SnO}}_{2}$ sensors relative to varying concentrations of ${\mathrm{SO}}_{2}$ “Reprinted (adapted) with permission from [103]. Copyright(2021) American Chemical Society”; (**iii**) schematic illustration of the sensing mechanism in the ${\mathrm{In}}_{2}$$O_{3}$/${\mathrm{TiO}}_{2}$ nanocomposites sensor activated by UV light at room temperature; (**iv**) response-time curves for ${\mathrm{In}}_{2}$$O_{3}$, ${\mathrm{TiO}}_{2}$, and ${\mathrm{In}}_{2}$$O_{3}$/${\mathrm{TiO}}_{2}$ nanocomposite sensors to ${\mathrm{CH}}_{2}$O concentrations ranging from 0.03 to 10 ppm; and (**v**) Nyquist plots elucidating the impedance response of the ${\mathrm{In}}_{2}$$O_{3}$/${\mathrm{TiO}}_{2}$ sensor to varying concentrations of ${\mathrm{CH}}_{2}$O “Reprinted (adapted) with permission from [104]. Copyright (2023) American Chemical Society”. (**D**) Schematic representation of the sensing mechanism in 0.01 wt% PANI-loaded 0.9-TiO_2_-0.1-NiO nanoparticles sensor depicting the different effects of NiO and PANI (**i**) without UV light, and (**ii**) with UV light; the selectivity of the sensor at various concentrations of acetone, benzene, toluene, CO, and ${\mathrm{NO}}_{2}$ at (**iii**) 300 °C without UV light and (**iv**) 25 °C with UV light (“Reprinted from [105], Copyright (2023), with permission from Elsevier”).

In addition to standalone pristine metal oxide gas sensors, the integration of nanostructured metal oxides to create heterojunction interfaces, such as n-type/n-type [93,94], p-type/p-type [95], n-type/p-type [96,97], and Schottky junctions [98], in well-defined core-shell and hierarchical nanocomposites, has synergistically enhanced room-temperature gas sensor performance through a light-activation strategy. Owing to their greater charge carrier mobility, MOs of n-type are generally favoured over p-type MOs [99]. However, p-type MOs have recently gained popularity in light-activated gas sensing because they offer advantages, such as lower operating temperatures and better humidity tolerance [100]. One innovative example of leveraging MOs heterostructures for gas sensing includes hollow ZnO-${\mathrm{TiO}}_{2}$ heterostructured nanospheres grown using a hydrothermal method with carbon nanospheres as sacrificial templates for formaldehyde (${\mathrm{CH}}_{2}$O) detection, as reported in [101]. When activated by a 365 nm UV LED, the ZnO-${\mathrm{TiO}}_{2}$-based chemi-resistive sensors showed a response of 11.02 to 10 ppm ${\mathrm{CH}}_{2}$O in ambient conditions, which was 5.48-fold higher than that of pristine hollow ZnO with reduced response and recovery times of 14 s and 16 s, respectively. This response improvement was attributed to the n-type carrier transfer from ZnO to ${\mathrm{TiO}}_{2}$, which equalized the Fermi levels and formed an accumulation layer of electrons on the ${\mathrm{TiO}}_{2}$’s surface, enhancing resistance modulation. Additionally, the self-constructed electric field at the ZnO/${\mathrm{TiO}}_{2}$ heterojunction increased the charge carrier separation efficiency (see Figure 6A(i)). These factors enabled the hollow ZnO/${\mathrm{TiO}}_{2}$ sensors to adsorb and react to formaldehyde more effectively, leading to a significantly improved response compared to pure ZnO sensors.

Comparative studies on UV-assisted gas-sensing setups show that heterojunctions and metal decoration significantly enhance sensing performance. In this context, Xu et al. explored the combined effects of heterojunction creation and metallic nanoparticles decoration to improve the gas-sensing capabilities of ${\mathrm{SnO}}_{2}$ modified with Ag nanoparticles (NPs) and polyaniline (PANI) for detecting sulphur dioxide (${\mathrm{SO}}_{2}$) at room temperature under UV light. They found that a response of 20.1 was nearly tenfold greater for 50 ppm of ${\mathrm{SO}}_{2}$ when compared to unmodified ${\mathrm{SnO}}_{2}$ [103]. This improved performance can be attributed to the generation of hot electrons through the localized surface plasmon resonance (LSPR) effect enabled by the Ag nanoparticles, which then migrate towards the ${\mathrm{SnO}}_{2}$ with the efficient carriers separation facilitated by the PANI/${\mathrm{SnO}}_{2}$ heterojunction. Similarly, Cai et al. demonstrated an enhanced ${\mathrm{NO}}_{2}$ sensor made possible by leveraging synergistic effects achieved through noble metal nanoparticle decoration and the formation of a heterojunction. By utilizing ${\mathrm{In}}_{2}$$O_{3}$ NPs decorated with Au NPs and embedded in ZnO porous nanofibers (Au-${\mathrm{In}}_{2}$$O_{3}$/ZnO) with 0.03 g of In salt, an enhanced response of 95.15 for 5 ppm concentration of ${\mathrm{NO}}_{2}$ under UV light at ambient temperature was reported [10]. This improvement was attributed to the improved density of the active site from the spillover effect and the formation of a Schottky barrier stimulated by gold nanoparticles and the Au-${\mathrm{In}}_{2}$$O_{3}$/ZnO heterostructure. Moreover, they observed that the hollow nanospheres (HNs) heterostructures of ${\mathrm{SnO}}_{2}$/${\mathrm{In}}_{2}$$O_{3}$ exhibited a response that was nearly 22 times higher than pristine ${\mathrm{SnO}}_{2}$ HNs when exposed to 100 ppm concentration of trimethylamine (TEA) gas under UV irradiation at room temperature with exceptional LOD of about 3.98 ppt. They attributed this significant enhancement to the existence of sub-grains and the formation of homo- and heterojunctions between ${\mathrm{SnO}}_{2}$, ${\mathrm{In}}_{2}$$O_{3}$ [102]. Further, Zhang et al. developed double-layer HNs of ${\mathrm{In}}_{2}$$O_{3}$/${\mathrm{TiO}}_{2}$, using a simple water-bath approach and carbon nanospheres as sacrificial templates. These nanocomposites, measuring between 150 and 250 nm with respective ${\mathrm{In}}_{2}$$O_{3}$ and ${\mathrm{TiO}}_{2}$ shell thicknesses of around 10 nm and 15 nm, demonstrated enhanced sensing capabilities for ${\mathrm{CH}}_{2}$O at room temperature under UV light [104] (see Figure 6C(iii,iv,v)). The heterostructured sensors exhibited an improvement of threefold in response to 1 ppm formaldehyde compared to pure ${\mathrm{In}}_{2}$$O_{3}$ sensors, with a notable response and recovery time of 28 s and 50 s, respectively. These improvements were attributed to the heterojunctions formed between ${\mathrm{In}}_{2}$$O_{3}$ and ${\mathrm{TiO}}_{2}$, which enhanced charge carrier separation and increased photocatalytic activity. Benefiting from the increased surface area and effective carrier transition from the core-shell structure, which provided redox reactions with more active locations, along with the potential barrier modulation enabled by the creation of a heterojunction in porous core-shell ${\mathrm{In}}_{2}$$O_{3}$/ZnO nanofibers (NFs), Min et al. demonstrated significantly enhanced gas-sensing abilities for toluene at room temperature, offering a response 6.4 times higher than pristine ${\mathrm{In}}_{2}$$O_{3}$ NFs [106]. In another work, Lee et al. demonstrated an enhanced gas sensor composed of NiO and ${\mathrm{TiO}}_{2}$ nanoparticle heterojunctions, synthesized via the sol–gel method and loaded with PANI, for detecting acetone at room temperature under UV light. They found that increasing the NiO content up to 0.01 wt% improved the sensor response, due to its catalytic properties, but that further increases diminished the response. UV illumination on PANI photogenerated electrons, which were transferred to ${\mathrm{TiO}}_{2}$, enhancing oxygen ion adsorption and carrier separation, due to the p–n junction formation (refer to Figure 6D(i,ii)). This setup allowed for the development of a low-power acetone sensor with notable stability and selectivity, applicable to similar sensor systems [105].

**Figure 7:**
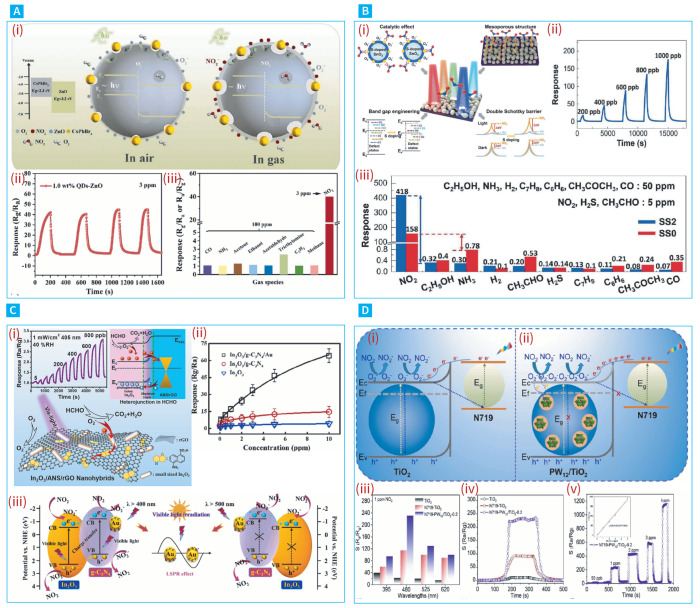
Gas sensors based on MOs heterostructures under visible-light activation: (**A**) (**i**) Illustration of the band diagram and gas-sensing mechanism in ZnO/CsPbBr3; (**ii**) the repeatability; (**iii**) the sensor selectivity to various gases (“Reprinted from [108], Copyright (2022), with permission from Elsevier”). (**B**) (**i**) Schematic illustration of band diagram and ${\mathrm{NO}}_{2}$ sensing mechanism under visible-light illumination in ${\mathrm{SnO}}_{2}$/S-${\mathrm{SnO}}_{2}$ nanoparticles; (**ii**) response toward 200–1000 ppb of ${\mathrm{NO}}_{2}$ with L-cysteine amount of 0.6 g (SS2); and (**iii**) selectivity of SS2 and SS0 (L-cysteine amount of 0 g) under blue-light illumination for various gases [111]. (**C**) (**i**) Enhanced sensing mechanism of ${\mathrm{In}}_{2}$$O_{3}$/ANS/rGO nanohybrids under visible-light illumination; the illustration shows the reaction of HCHO molecules on the surface of the nanohybrids, with the conversion of $O_{2}$ and HCHO into ${\mathrm{CO}}_{2}$ and $H_{2}$O facilitated by changes in the electrical double layer (EDL) thickness and a potential barrier at the ${\mathrm{In}}_{2}$$O_{3}$ homojunction, which occurs both in air and in the presence of HCHO; energy band diagram of InAG heterojunction in HCHO atmosphere (inset right) and response profiles of the InAG sensor to varying concentrations of HCHO at room temperature (inset left) “Reprinted (adapted)with permission from [112]. Copyright (2023) American Chemical Society”; (**ii**) fitted responses of the sensors in [113] as a function of ${\mathrm{NO}}_{2}$ concentration from 0.02 to 10 ppm at room temperature with visible-light activation; and (**iii**) schematic representation of the enhanced ${\mathrm{NO}}_{2}$ sensing mechanism by ${\mathrm{In}}_{2}$$O_{3}$/g-$C_{3}$$N_{4}$/Au NFs under visible light (>400 nm and >500 nm) and the corresponding energy band diagrams (“Reprinted from [113], Copyright (2022), with permission from Elsevier”). (**D**) Schematic depiction of the sensing mechanisms for (**i**) N719-${\mathrm{TiO}}_{2}$ films and (**ii**) N719-PW12/${\mathrm{TiO}}_{2}$ films in [115] under light illumination. The blue, green, and orange spheres denote ${\mathrm{TiO}}_{2}$, N719, and PW12 molecules, respectively; (**iii**) comparison of responses for the sensors to 1 ppm ${\mathrm{NO}}_{2}$ under different light wavelengths; (**iv**) response and recovery curves of the sensors to 1 ppm ${\mathrm{NO}}_{2}$; (**v**) dynamic response for the PW12/${\mathrm{TiO}}_{2}$ sensor under 480 nm light illumination (“Reprinted from [115], Copyright (2023), with permission from Elsevier”).

As can be inferred from the reviewed works above, UV illumination is preferable in MOs-based light-assisted gas sensing, due to the wide bandgap of these oxides. However, extended UV exposure can lead to health risks and sample deterioration. This underscores the need for gas sensors that function under visible light. However, the inherent transparency of these wide-bandgap MOs to visible light presents a significant obstacle. Various techniques have been proposed to overcome this, such as employing the localized surface plasmon resonance (LSPR) effect of plasmonic noble metal nanoparticles and using narrow-bandgap photosensitizers that can absorb visible light to generate photo carriers. In this context, Huang et al. demonstrated the use of copper phthalocyanine (CuPc) as a photosensitizer to extend light absorption into the visible region, synthesizing a CuPc/ZnO nanocomposite via a one-step microwave-assisted hydrothermal method. This composite showed an improved response to 80 ppm of ammonia (${\mathrm{NH}}_{3}$) under red light at room temperature, with a rapid response time of 20 s and a recovery time of 10 s with good humidity resistance and a low detection threshold of 0.8 ppm [107]. Moreover, Yueyue et al. took advantage of the effective transfer and reduced recombination of charge carriers facilitated by the heterojunction between the CsPbBr3 quantum dots (QDs) that exhibited p-type semiconductor properties and ZnO microballs (MBs) (see Figure 7A(i)), to enhance the sensing of ${\mathrm{NO}}_{2}$ [108]. In an ambient environment and under 520 nm visible light illumination, the optimized composite, containing 1 wt% QDs, achieved a notable response of 53 to 5 ppm of ${\mathrm{NO}}_{2}$ with excellent selectivity (Figure 7A(ii,iii)). On the other hand, in pursuit of addressing the common challenges of low response and stability in MOs methane sensors, Zhang et al. employed the small bandgap carbon nitride ZnO/g-$C_{3}$$N_{4}$ semiconductor as a photosensitizer, to enhance the methane-sensing performance of ZnO/g-$C_{3}$$N_{4}$ composites under visible light activation at room temperature [109]. The ZnO/g-$C_{3}$$N_{4}$ composite with 5 wt% g-$C_{3}$$N_{4}$ demonstrated a significantly improved response of 6.89 to 1000 ppm ${\mathrm{CH}}_{4}$, owing to efficient charge carrier separation at the heterojunction interface. The ${\mathrm{CO}}_{2}$ sensing of ${\mathrm{SnO}}_{2}$ decorated with CdO heterostructures under visible light illumination was studied by Singh et al. [110], who prepared the heterostructures following a one-step hydrothermal method and reported a 2.6-fold higher sensing performance under visible light assistance compared to dark conditions for 200 ppm ${\mathrm{CO}}_{2}$, due to the effective separation of the heterojunction photogenerated carriers, which facilitated the photo-adsorption and photo-desorption processes of the $O_{2}$ and ${\mathrm{CO}}_{2}$ molecules. In another study, Eom et al. developed a sulphur-doped (S-${\mathrm{SnO}}_{2}$) sensor, using a hydrothermal method with L-cysteine as a sulphur doping agent, demonstrating a notable response of 418 and rapid recovery and response times of 64 s and 170 s, respectively, towards 5 ppm of ${\mathrm{NO}}_{2}$ under blue-light irradiation [111]. The incorporation of sulphur introduced defect states within the bandgap, effectively narrowing it and enhancing visible light absorption. Additionally, the porous structure and large surface area of the S-${\mathrm{SnO}}_{2}$ further improved its gas-sensing performance, making it plausible for indoor air quality monitoring in the context of health-related applications. Furthermore, Guo et al. reported the efficacy of visible-light-driven heterojunctions for enhancing gas-sensing performance in ambient conditions. They utilized ultrasmall ${\mathrm{In}}_{2}$$O_{3}$ nanorods (NRs) assembled with 5-aminonaphthalene-1-sulfonic acid (ANS) and supramolecularly functionalized reduced graphene oxide to create a hybridized heterojunction with InAG sensors [112]. These sensors showed a remarkable response of 2.4 towards 0.5 ppm of formaldehyde under 405 nm illumination, being 1.7 and 1.3 times greater than the ${\mathrm{In}}_{2}$$O_{3}$ pristine NRs and ${\mathrm{In}}_{2}$$O_{3}$/rGO, respectively, with a 5 ppb limit of detection. The enhanced response was ascribed to the large-area heterojunctions promoting efficient separation and the extended lifetime of the photogenerated charge carriers, thereby ensuring high selectivity and stability for indoor formaldehyde detection at ppb levels. The synergistic effects of heterojunction formation and plasmonic metal decoration on improving gas-sensing performance under visible illumination at room temperature have been explored by Han et al. [113]. They fabricated ${\mathrm{In}}_{2}$$O_{3}$/g-$C_{3}$$N_{4}$-Au heterojunction nanofibers, using methods of electrospinning, vapor deposition, and in situ reduction. The ${\mathrm{In}}_{2}$$O_{3}$/g-$C_{3}$$N_{4}$-Au sensors exhibited a superior response of 17.2 towards 1 ppm ${\mathrm{NO}}_{2}$, significantly outperforming the pristine ${\mathrm{In}}_{2}$$O_{3}$ and ${\mathrm{In}}_{2}$$O_{3}$/g-$C_{3}$$N_{4}$ by factors of 6.8 and 2.2, respectively. This enhancement was attributed to the combined effects of the Au nanoparticles, which increased the active sites and carrier density through the LSPR and Schottky barrier effects, alongside their catalytic properties. Furthermore, this ternary heterojunction facilitated the efficient separation of photogenerated carriers, thereby improving the sensor’s overall performance under visible-light illumination. The improvement of $H_{2}$ sensing utilizing the establishment of nano-Schottky junctions between Pd and ${\mathrm{TiO}}_{2}$ and p-n heterojunctions between PdO and ${\mathrm{TiO}}_{2}$ was also demonstrated by Thathsara et al. [114]. The formed nano-Schottky junctions effectively hindered the recombination of charge carriers. Under 565 nm visible-light illumination at ambient temperature, an $H_{2}$ response of 100.82% at 500 ppm was recorded, with response and recovery times of 77 s and 470 s, respectively.

Moreover, Sun et al. demonstrated that incorporating polyoxometalate (POM) into N719/${\mathrm{TiO}}_{2}$ films improves the ${\mathrm{NO}}_{2}$-sensing abilities of visible-light-activated ${\mathrm{TiO}}_{2}$-based gas sensors operating at room temperature [115]. The POM/N719/${\mathrm{TiO}}_{2}$ composite films, when illuminated with 480 nm light, revealed a superior response of 231 and reduced response/recovery times of 48 s/66 s for a concentration of 1 ppm ${\mathrm{NO}}_{2}$, owing to the POM’s role as an electron scavenger, facilitating rapid carrier separation and efficient electron transfer within the structure, leading to heightened sensitivity and faster detection compared to both pristine ${\mathrm{TiO}}_{2}$ and N719/${\mathrm{TiO}}_{2}$ thin films. In another study, Liu et al. demonstrated that a room-temperature, visible-light-illuminated gas sensor, featuring a hierarchical porous ${\mathrm{WO}}_{3}$/${\mathrm{CuWO}}_{4}$ heterostructure, exhibited enhanced sensitivity, with a response value of 82 towards 1 ppm ${\mathrm{NO}}_{2}$ [116]. This performance was substantially higher—8.2, 12.6, and 3.5 times—compared to sensors made of pristine ${\mathrm{WO}}_{3}$, ${\mathrm{CuWO}}_{4}$, and a combination of ${\mathrm{WO}}_{3}$/${\mathrm{CuWO}}_{4}$ nanoparticles, respectively. The notable increase in sensitivity was attributed to the effective extraction of photogenerated charge carriers facilitated by the heterostructure, alongside enhanced gas adsorption due to the sensor’s unique porous framework.

### 2.4. Optically Activated Gas Sensors Based on TMDCs

Transition metal dichalcogenides (TMDCs) are semiconducting materials composed of a transition metal *M* (such as Mo or W) and a chalcogen element *X* (S or Se) in the $MX_{2}$ structure. These materials are attracting considerable attention from researchers as a promising alternative to graphene within the field of two-dimensional materials [117,118]. TMDCs include compounds like molybdenum disulfide (${\mathrm{MoS}}_{2}$), molybdenum diselenide (${\mathrm{MoSe}}_{2}$), tungsten diselenide (${\mathrm{WSe}}_{2}$), tungsten disulfide (${\mathrm{WS}}_{2}$), and molybdenum ditelluride (${\mathrm{MoTe}}_{2}$). As their layer number decreases from several layers to a monolayer, they transition from being indirect bandgap semiconductors to direct bandgap semiconductors and exhibit fascinating optoelectronic properties. They are also highly esteemed options for flexible electronics, due to their excellent charge-transport properties, absence of dangling bonds on the surface, and compatibility with biological systems. In particular, ${\mathrm{MoS}}_{2}$ possesses numerous beneficial attributes that render it ideal for gas-molecule adsorption. These include rapid charge transfer, adaptable optical properties, a tunable band gap, exceptional carrier mobility, significant reactivity, and, notably, a substantial surface-to-volume ratio [119,120].

**Figure 8:**
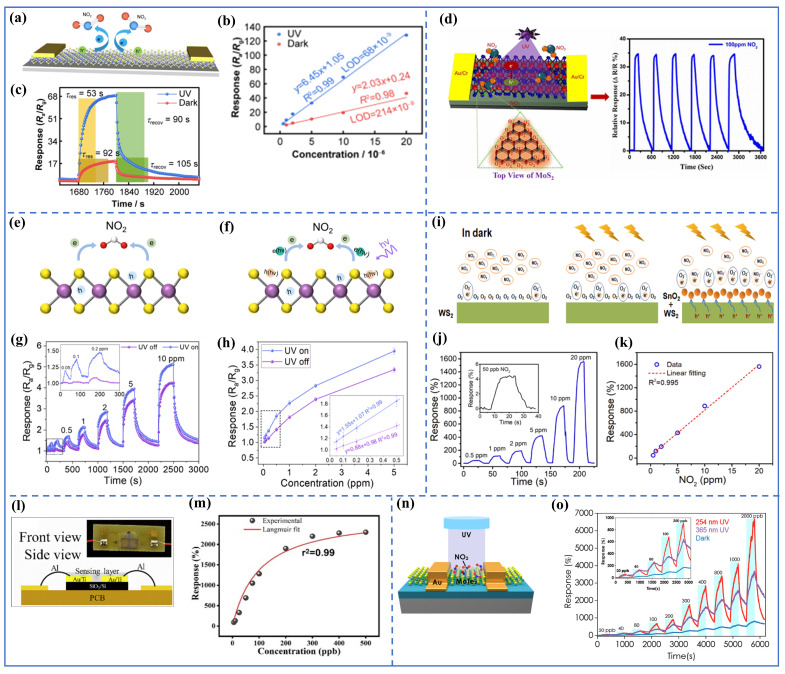
Gas sensors based on TMDC 2D nanomaterials driven by optical sources: (**a**) Schematic illustration of sensing mechanism of monolayer (1 L) ${\mathrm{WSe}}_{2}$ sensor to ${\mathrm{NO}}_{2}$; (**b**) the performance of 1 L ${\mathrm{WSe}}_{2}$ gas sensor for ${\mathrm{NO}}_{2}$ at RT; and (**c**) its response fitting curves of different ${\mathrm{NO}}_{2}$ concentrations without and with UV-light irradiations; under UV illumination, the sensor’s response to 1 × $10^{-6}$${\mathrm{NO}}_{2}$ at room temperature (25 °C) reaches up to 9, marking a fourfold enhancement compared to the sensor’s response without UV activation [121]; (**d**) schematic illustration of ${\mathrm{MoS}}_{2}$-based sensor, and its relative response under UV light [122]; schematic sensing process of ${\mathrm{WSe}}_{2}$ nanosheets in response to ${\mathrm{NO}}_{2}$ (**e**) without and (**f**) with UV illumination; (**g**) the sensing curve to ${\mathrm{NO}}_{2}$ of different concentrations without and with UV illumination; (**h**) the sensor response of ${\mathrm{WSe}}_{2}$ nanosheets to ${\mathrm{NO}}_{2}$ with increasing concentration without and with UV illumination; the inset is the linear fit of the sensor response to low ${\mathrm{NO}}_{2}$ concentration [123]; (**i**) schematic sensing mechanisms of ${\mathrm{WS}}_{2}$ and ${\mathrm{WS}}_{2}$/${\mathrm{SnO}}_{2}$ heterojunction without and with UV illumination; (**j**) dynamic response curves; and (**k**) responses and linear fitting curve of ${\mathrm{WS}}_{2}$/${\mathrm{SnO}}_{2}$ sensor to 0.5–20 ppm ${\mathrm{NO}}_{2}$ at RT under UV illumination [124]; (**l**) the ${\mathrm{MoS}}_{2}$/ZnO-based actual device attached and wire-bonded with a printed circuit board (PCB); and (**m**) the response plotted against the concentration of ${\mathrm{NO}}_{2}$ between 5 and 500 ppb and a fitted curve based on the Langmuir adsorption isotherm [125]; (**n**) schematic diagram of ${\mathrm{MoTe}}_{2}$-based gas sensor; and (**o**) its dynamic sensing performance with and without UV illumination. The inset shows the dynamic sensing behaviors to ${\mathrm{NO}}_{2}$ at concentration ranging from 20 to 200 ppb. The light-blue bars represent the concentration of gas for each instance of exposure [126].

While considerable strides have been made in leveraging TMDCs for these purposes, challenges must be overcome, to further progress in this field. Therefore, employing an optical source in active gas sensor devices is a predetermined solution for enhancing sensitivity and achieving fast response/recovery times. The interaction between gas molecules and the sensor material can induce rapid changes in optical properties, which can be detected almost instantaneously. Consequently, interest in optically activated gas sensors based on TMDCs has been increasing in recent years. Guo-Cai Lu et al. reported a highly sensitive ${\mathrm{NO}}_{2}$ sensor based on a ${\mathrm{WSe}}_{2}$ monolayer, which exhibited fourfold enhancement in response to ${\mathrm{NO}}_{2}$ under UV illumination compared to conditions without UV activation [121]. As shown in Figure 8a, the ${\mathrm{WSe}}_{2}$-based sensor achieved a lower limit of detection of 0.068 ppm under UV light compared to 0.214 ppm in the dark (see Figure 8b). Additionally, Figure 8c reveals a comparison of the response and recovery times of the ${\mathrm{WSe}}_{2}$ gas sensor. The results indicate that when exposed to UV irradiation, the sensor exhibits shorter response (53 s) and recovery times (90 s) compared to operation in the dark. This improvement can be attributed to the higher energy of UV light, which exceeds the band gap energy of ${\mathrm{WSe}}_{2}$, leading to the generation of a larger number of photogenerated charge carriers. This process enhances the adsorption and desorption processes of gas molecules, thereby improving the sensor’s overall response and recovery performance.

Additionally, Rahul Kumar et al. demonstrated a reversible ${\mathrm{MoS}}_{2}$ gas sensor capable of rapid detection at room temperature (RT). The gas sensor’s performance was evaluated for ${\mathrm{NO}}_{2}$ gas under thermal and photoactivated conditions. It was shown that the sensing device exhibited a high response time of approximately 249 s and partial recovery at RT. While the utilization of thermal energy was adequate for achieving full recovery, it came at the expense of reduced sensitivity. However, under photoexcitation, ${\mathrm{MoS}}_{2}$ demonstrated heightened sensitivity, with a quick response time of around 29 s, and remarkable recovery characteristics toward 100 ppm ${\mathrm{NO}}_{2}$, as depicted in Figure 8d. The observed enhancement in sensitivity and response time can be attributed to changes in the charge distribution on the sensing surface during the interaction between ${\mathrm{NO}}_{2}$ and ${\mathrm{MoS}}_{2}$, especially under optical illumination [122]. Another study by Chen Yang et al. demonstrated a gas sensor with high sensitivity and selectivity, using a ${\mathrm{WSe}}_{2}$ nanosheet for detecting ${\mathrm{NO}}_{2}$ under UV activation at RT [123]. Here, the impact of UV exposure on sensing capabilities was thoroughly examined. The results from Figure 8g,h indicate a significant improvement in sensing ${\mathrm{NO}}_{2}$ upon UV irradiation, with negligible effects observed on reducing gases. This enhancement led to an exceptionally low detection limit of 8.0 ppb for ${\mathrm{NO}}_{2}$ detection.

The response time of TMDC 2D layers/quantum dot heterostructures enhances the gas sensor’s response time under UV illumination. Yi Xia et al. achieved rapid response and recovery times along with complete reversibility when detecting ${\mathrm{NO}}_{2}$ at RT by simply decorating ${\mathrm{WS}}_{2}$ nanosheets with ${\mathrm{SnO}}_{2}$ quantum dots under UV illumination [124]. As can be seen from Figure 8j,k, the response/recovery time was achieved around 9 s/8 s within a detection range of 0.5–20 ppm. Here, UV-light illumination significantly increased the sensitivity of the sensors, highlighting the crucial role of UV activation in improving response/recovery rates because of the efficient interfacial charge separation in the heterostructures.

Furthermore, Rishi Ranjan Kumar and his colleagues reported a high-performance ${\mathrm{NO}}_{2}$ gas sensor, using an ${\mathrm{MoS}}_{2}$/ZnO nanohybrid at RT. Under UV activation, the nanohybrid sensor exhibited remarkable 91% and 2310% responses at 5 and 500 ppb ${\mathrm{NO}}_{2}$, respectively (see Figure 8l,m). It should be noted that the sensor response was calculated using the formula $s\left(\%\right)=\left(R_{g}-R_{a}\right)/R_{a}\times 100\%$, where $R_{a}$ was the resistance in air and $R_{g}$ the resistance in the target gas (${\mathrm{NO}}_{2}$). Additionally, the low concentration sensitivity and the lowest detection limit were estimated to be around 0.135 ${\mathrm{ppb}}^{-1}$ and 0.2 ppb, respectively [125]. The sensor’s remarkable responses to ${\mathrm{NO}}_{2}$ were attributed to the synergistic effects of ${\mathrm{MoS}}_{2}$ and ZnO. Despite the slower response and recovery times, achieving an extremely low detection limit of ${\mathrm{NO}}_{2}$ was possible using pristine TMDC layers under UV illumination. As shown in Figure 8n,o, an ultrasensitive p-type ${\mathrm{MoTe}}_{2}$ gas sensor for ${\mathrm{NO}}_{2}$ detection with greatly enhanced sensitivity and recovery rate under UV illumination was reported. The calculated limit of detection for ${\mathrm{NO}}_{2}$ gas was found to be 252 parts per trillion (ppt) under 254 nm UV illumination [126]. Such remarkable characteristics stem from the activated interaction at the interface between ${\mathrm{NO}}_{2}$ gas molecules and the p-type ${\mathrm{MoTe}}_{2}$ surface when exposed to UV light. While the limit of detection is lower compared to other reports, ${\mathrm{MoS}}_{2}$ can be further enhanced by combining it with oxides, to improve gas-sensing properties. For instance, He et al. established an ${\mathrm{SnO}}_{2}$/${\mathrm{MoS}}_{2}$ sensor capable of detecting ${\mathrm{SO}}_{2}$ gas under UV irradiation. The response of this device was 4.68 with an LOD of 1 ppm, and it exhibited good selectivity, repeatability, and stability [127].

Furthermore, TMDCs can be designed to be highly selective to specific gases, minimizing interference from other substances. When hybridized with other materials, such as oxides, their synergistic effects further enhance their gas-sensing abilities. The implications of these properties are profound, with potential applications in industrial process monitoring and environmental monitoring. Notably, their tunable bandgaps and high surface-to-volume ratio, which increases with decreasing layer thickness, make them uniquely suited to enhanced sensitivity and selectivity. Moreover, their layered structure enables precise control over electronic properties, allowing for tailored detection of specific gases. Unlike metal oxides, which often require high operating temperatures, TMDCs can operate effectively at room temperature, making them more energy-efficient. Additionally, compared to materials like graphene, which lacks a bandgap, TMDCs provide superior control over sensing mechanisms. Consequently, their unique electronic, optical, and structural properties position TMDCs as premier materials for developing next-generation gas sensors that offer exceptional sensitivity, selectivity, and flexibility.

## 3. Conclusions and Outlook

### 3.1. Conclusions

Recent advancements in gas sensor technology have demonstrated significant progress. Particularly with the incorporation of 2D nanomaterials, metal oxides, and organic materials as active layers: these materials are leading the way in gas sensing, due to their unique properties and wide-ranging applications. Table 1 highlights their exceptional performance in detecting gases, such as ${\mathrm{NO}}_{2}$, ${\mathrm{NH}}_{3}$, $H_{2}$, ${\mathrm{CO}}_{2}$, and ${\mathrm{Cl}}_{2}$.

Graphene-based gas sensors have emerged as a significant advancement in the field of gas detection, offering a unique combination of high sensitivity, rapid response times, and the potential for miniaturization and integration into various platforms. Moreover, functionalization techniques, including the attachment of metal oxides, metal nanoparticles, and organic molecules, have significantly improved the selectivity of graphene-based sensors, enabling them to distinguish between target gases and potential interferents. These advancements underscore the potential of graphene-based gas sensors to revolutionize applications in environmental monitoring, industrial safety, and healthcare diagnostics. Despite these promising developments, several challenges remain. Achieving consistent selectivity across various gases and ensuring long-term stability and reproducibility of sensor responses are critical hurdles. Additionally, the integration of graphene-based sensors into commercial devices and systems poses technical and economic challenges that need to be addressed for widespread adoption.

The exploration of optically activated gas sensors based on metal oxides (MOs) has demonstrated significant advancements in enhancing gas-detection capabilities through photoactivation. Various studies have highlighted the role of heterojunctions, noble metal decorations, and the unique structural properties of these materials in further boosting their performance. The integration of heterostructured nanomaterials has enabled better charge carrier separation, enhanced redox reactions, and increased active sites for gas interactions, leading to significant improvements in sensor response, stability, and selectivity.

Organic materials, while offering advantages, like ease of synthesis and room temperature operation, face limitations, such as poor stability, low surface area, and reduced sensitivity. However, hybrid organic–inorganic sensors have emerged as a viable solution, combining the benefits of organic materials with the enhanced stability and sensitivity of inorganic components. The introduction of metal–organic frameworks (MOFs) marked a significant advancement in this field. MOFs, characterized by high porosity, large specific surface area, and versatile structures, have demonstrated exceptional gas-sensing abilities. Various studies have shown that MOFs can be tailored for specific gas detection, providing high sensitivity and fast response times. Organic conducting polymers (CPs) have also been explored for gas-sensing applications, with materials like polypyrrole (PPy) and polyaniline (PANI) showing promise. These polymers offer high sensitivity and mechanical robustness, making them suitable for various sensing applications. Innovations in sensor design, such as the use of fiber-optic systems and surface plasmon resonance (SPR) sensors, have further enhanced the performance and functionality of organic-based gas sensors. Moreover, the use of organic polymers in optical-fiber Fabry–Perot (F–P) cavities has demonstrated the potential for high-precision gas measurements, particularly in detecting acid gases. This method, along with integrated optical interferometers, has paved the way for the development of sensitive and selective gas sensors. Overall, the advancements in hybrid organic–inorganic materials, including MOFs and CPs, have significantly enhanced the abilities of optically activated gas sensors. Despite the challenges associated with pure organic materials, ongoing research and development continue to improve their performance, making them viable candidates for practical applications. This review underscores the potential of these materials in gas-sensing technologies and highlights the need for continued exploration, to overcome existing limitations and unlock new possibilities in the field.

Finally, we conclude by highlighting the potential of TMDCs as optically activated gas sensors. As detailed in the discussion, materials like ${\mathrm{MoS}}_{2}$, ${\mathrm{WSe}}_{2}$, and ${\mathrm{MoTe}}_{2}$ have demonstrated unique properties that make them highly suitable for enhanced gas-sensing applications, particularly under optical illumination, such as UV light. This enhancement is primarily attributed to the increased generation of photogenerated charge carriers, which facilitates more efficient adsorption and desorption of gas molecules. For instance, ${\mathrm{MoS}}_{2}$ sensors display heightened sensitivity and faster recovery under photoexcitation conditions. Additionally, the incorporation of TMDCs with other materials, such as ZnO and ${\mathrm{SnO}}_{2}$, has led to the development of hybrid sensors with even more remarkable properties. These hybrids leverage the synergistic effects of their constituent materials, resulting in lower detection limits and enhanced response characteristics, which are crucial for advancing the field of optical gas sensing. In addition to TMDCs, another interesting material is Mxene. There have been significant advancements in the development of Mxene-based sensors over the past decade, with numerous comprehensive reviews underscoring this progress [131,132,133,134,135]. However, due to the semi-metallic nature and the nearly zero band gap of Mxene, it is unsuitable for use as a standalone active material. Instead, it is typically employed in conjunction with other active materials within a hybrid structure. Consequently, Mxene has not been extensively explored for use in optical-based gas-sensing applications.

### 3.2. Future Outlook

The future of optically activated gas sensors based on 2D materials is promising, offering significant potential for advancement and application. Several key areas for future research and development can be identified:

1. Enhanced sensing performance: There is a need to further improve the sensitivity, selectivity, and response times of transition metal dichalcogenide (TMDC)-based optically activated gas sensors. Achieving this may involve innovative material synthesis methods and optimizing device structures. For instance, the optical properties of TMDCs can be harnessed for gas sensing, as interactions between gas molecules and the TMDC surface induce measurable changes in the material’s optical characteristics, such as photoluminescence (PL) intensity, Raman shifts, or absorption spectra. Although research on cavity-enhanced light–matter interactions in TMDC monolayers, particularly with strongly bound excitons for optically assisted gas sensing, is limited, integrating TMDCs with plasmonic cavities offers significant advantages [136,137]. This integration enhances the electromagnetic field near the TMDC surface, resulting in a stronger PL response when gas molecules interact with the TMDC layer. Consequently, plasmonic cavities can amplify even subtle changes in PL intensity, enabling the detection of low concentrations of gas molecules with greater precision. Hybrid TMDC–plasmonic cavity sensors are highly effective for environmental monitoring, particularly for detecting and quantifying trace gases like nitrogen dioxide (${\mathrm{NO}}_{2}$) and sulfur dioxide (${\mathrm{SO}}_{2}$) in the atmosphere [7]. The enhanced sensitivity and selectivity of these sensors result from leveraging the strong light–matter interactions provided by the plasmonic cavities, which amplify the TMDCs’ response to specific gas molecules. Furthermore, dielectric metasurfaces with a high Q-factor are highly desirable for enhancing the performance of sensing [138]. Hybridizing these high Q-factor dielectric metasurfaces with ultrathin layers of TMDCs can further increase sensitivity to minute environmental changes, making for real-time sensing. Despite notable progress having been made in sensitivity enhancement and detection limit refinement, achieving superior selectivity remains a significant challenge. Subsequently, future endeavours should concentrate on crafting selective sensing materials and investigating advanced signal-processing techniques, to distinguish target gases from interfering elements effectively.
2. Integration with IoT and smart systems: Integrating TMDC-based optically activated gas sensors into Internet of Things (IoT) platforms and smart systems facilitates real-time gas concentration monitoring and data analysis. This integration enables remote surveillance of environmental pollutants and dangerous gases, empowering timely responses to potential hazards. Future research should emphasize scalable fabrication methods and energy-efficient designs, to enable the widespread deployment of smart sensing networks, bolstering environmental and public-health-monitoring abilities.
3. Multi-gas detection: There is a need to develop novel optically activated gas sensors capable of simultaneously detecting multiple gases. This multi-gas detection capability enhances sensor versatility and utility across various applications. In addition to detecting different gases, it is important to separate their individual concentrations. In this respect, artificial intelligence could be used to train sensors to distinguish different gases and measure their concentrations.
4. Miniaturization and wearable devices: Shrinking the size of TMDC-based optically activated gas sensors allows integration into wearable devices for personal exposure monitoring and health tracking. These compact, portable sensors furnish individuals with real-time insights into their immediate gas surroundings.
5. Environmental and industrial applications: TMDC-based optically activated gas sensors offer substantial promise for environmental monitoring and industrial safety. Optically activated gas sensors based on graphene and metal oxides have shown significant promise in environmental monitoring [12], particularly in detection of disease-related volatile organic compounds (VOCs) by means of nanomaterial-based sensors. A notable example is the use of these sensors in smart city projects, where continuous monitoring of air quality is very important [13]. Future research should involve field deployment, to assess sensor performance in diverse conditions, validating reliability and durability. Additionally, efforts towards reducing the operating temperatures of metal oxides-based gas sensors, enhancing response/recovery times, and integrating energy-efficient sensing mechanisms (such as photoactivation) could further expand their applicability in diverse environmental and industrial settings.
6. Multifunctional hybrid materials: Exploring the integration of multiple 2D nanomaterials and hybrid structures could yield multifunctional gas sensors with improved performance and adaptability. Incorporating novel materials like metal–organic frameworks (MOFs), Mxene, and quantum dots may facilitate tailored sensing platforms for a wide array of gas analytes. The integration of organic materials with inorganic components, particularly in the form of hybrid sensors, is likely to play a crucial role in overcoming current challenges, such as stability, surface area limitations, and sensitivity at room temperature.
7. Flexible and wearable sensors: Responding to the burgeoning demand for wearable and flexible electronics, the development of lightweight, bendable gas sensors based on 2D nanomaterials presents promising opportunities in environmental monitoring, healthcare, and personal safety. Future research should prioritize optimizing sensor design and fabrication processes, to bolster durability, sensitivity, and user comfort.
8. Improved detection capability: Organic materials, such as polymers and metal–organic frameworks (MOFs), offer advantages, such as tunable chemical functionalities, high surface areas, and biocompatibility. In the future, these materials are expected to continue evolving towards enhanced stability, selectivity for specific gases, and integration into flexible and wearable sensor platforms. Applications could expand into areas requiring sensitive detection of volatile organic compounds (VOCs), bioanalytes, and environmental pollutants with improved sensing capabilities and reliability.

## Figures and Tables

**Table 1:** Lists the gas sensors featuring an active layer based on 2D nanomaterials. The calculation of sensor sensitivity can be explained according to the nature of the semiconductor and the relative change in resistance [128]. As a result, the response in the references was calculated as follows: for ^*a*^, the formula was $R=R_{g}/R_{a}$; for ^*b*^, the formula was $R=R_{a}/R_{g}$, and for ^*c*^, the formula was $R=\frac{R_{g}-R_{a}}{R_{a}}\times 100\;\left(\%\right)$.

Active Material	Active Layer	Gas	Conc. (ppm)	Response	LOD (ppm)	Res./Recovery Time(s)	Ref
Graphene	Graphene/${\mathrm{MoS}}_{2}$	${\mathrm{NO}}_{2}$	10	${0.69\;}^{c}$	0.2	0.7/0.9	[38]
	Graphene	${\mathrm{CO}}_{2}$	-	-	$1.4\times 10^{-4}$	-	[33]
	Graphene	${\mathrm{NO}}_{2}$	-	-	$5.2\times 10^{-4}$	-	[33]
	Graphene	${\mathrm{CH}}_{4}$	-	-	$7.2\times 10^{-4}$	-	[33]
	Pd-doped rGO + ZnO-${\mathrm{SnO}}_{2}$	$H_{2}$	100	${9.4\;}^{b}$	0.047	4/8	[39]
	Multilayer graphene	${\mathrm{NO}}_{2}$	1	${3.1\%\;}^{c}$	0.03	330/-	[23]
	rGO/cpoPcCo	${\mathrm{NH}}_{3}$	100	${42.4\;}^{c}$	3.7	120	[43]
TMDC	${\mathrm{WS}}_{2}$/${\mathrm{SnO}}_{2}$ heterostructures	${\mathrm{NO}}_{2}$	0.5	${51\%\;}^{c}$	0.05	9/8	[124]
	P-type ${\mathrm{MoTe}}_{2}$	${\mathrm{NO}}_{2}$	1	${1300\%\;}^{c}$	$2.52\times 10^{-4}$	300/160	[126]
	Single-layer ${\mathrm{WSe}}_{2}$	${\mathrm{NO}}_{2}$	5	${35\;}^{b}$	0.068	76/109	[121]
	${\mathrm{WSe}}_{2}$ Nanosheets	${\mathrm{NO}}_{2}$	10	${5.36\;}^{b}$	0.008	175/241	[123]
	${\mathrm{MoS}}_{2}$/ZnO nanohybrid	${\mathrm{NO}}_{2}$	100	${91\%\;}^{c}$	$2.0\times 10^{-4}$	130/110	[125]
	${\mathrm{MoS}}_{2}$ + ${\mathrm{Ti}}_{3}$$C_{2}$$T_{x}$ MXene	${\mathrm{NH}}_{3}$	100	${81.7\%\;}^{c}$	0.2	3/2.4	[129]
	${\mathrm{MoS}}_{2}$ Nanoflakes	${\mathrm{NO}}_{2}$	3	${2.82\%\;}^{c}$	0.19	9/3	[130]
Organic	ZIF-8	${\mathrm{CO}}_{2}$	-	${98\%\;}^{c}$	-	9	[50]
	ZIF-8	Acetone	62	-	6.67	1/5	[51]
	PR-MOF-1	EDA	0.001	${3.45\;}^{a}$	-	2	[53]
	PANI/GNF	${\mathrm{NH}}_{3}$	0.125	-	0.04	381/406	[58]
Metal oxide	${\mathrm{TiO}}_{2}$	${\mathrm{NO}}_{2}$	5	12,200% ^*c*^	$0.202\times 10^{-3}$	428	[73]
	ZnO	$C_{2}$$H_{5}$OH	1000	${45\;}^{b}$	10	6/94	[79]
	${\mathrm{In}}_{2}$ $O_{3}$	${\mathrm{NO}}_{2}$	0.8	14.9 ^*b*^	0.1	14/67	[85]
	${\mathrm{In}}_{2}$ $O_{3}$	$H_{2}$S	1	26,268.5 ^*b*^	0.001	48	[86]
	ZnO/${\mathrm{TiO}}_{2}$	HCHO	10	11.2 ^*b*^	5	14/16	[101]
	Au-${\mathrm{In}}_{2}$$O_{3}$/ZnO	${\mathrm{NO}}_{2}$	5	95.15 ^*a*^	0.05	119/377	[10]
	${\mathrm{SnO}}_{2}$/${\mathrm{In}}_{2}$$O_{3}$	Triethylamine	100	34 ^*b*^	$3.98\times 10^{-6}$	200/500	[102]
	${\mathrm{In}}_{2}$$O_{3}$/${\mathrm{TiO}}_{2}$	HCHO	1	3.8 ^*b*^	0.06	28/50	[104]
	${\mathrm{In}}_{2}$$O_{3}$/ZnO	Toluene	100	34.7 ^*b*^	0.1	213/1709	[106]
	PANI/NiO-loaded ${\mathrm{TiO}}_{2}$	acetone	50	11.3 ^*b*^	0.176	150/290	[105]
	CuPc/ZnO	${\mathrm{NH}}_{3}$	80	15.8 ^*b*^	0.8	20/10	[107]
	ZnO/${\mathrm{CsPbBr}}_{3}$	${\mathrm{NO}}_{2}$	5	53 ^*a*^	0.041	63/40	[108]
	${\mathrm{SnO}}_{2}$/S-${\mathrm{SnO}}_{2}$	${\mathrm{NO}}_{2}$	5	418 ^*a*^	$9\times 10^{-7}$	170/64	[111]
	${\mathrm{In}}_{2}$$O_{3}$/ANS/rGO nanohybrids	HCHO	0.5	2.4 ^*b*^	0.005	19/179	[112]
	${\mathrm{In}}_{2}$$O_{3}$/g-$C_{3}$$N_{4}$-Au	${\mathrm{NO}}_{2}$	1	17.2 ^*a*^	0.020	-	[113]
	PdO/${\mathrm{TiO}}_{2}$	$H_{2}$	500	100.29% ^*c*^	2.03	77/470	[114]
	N719-sensitized POM/${\mathrm{TiO}}_{2}$ films	${\mathrm{NO}}_{2}$	1	231 ^*b*^	0.05	48/66	[115]
	hierarchical porous ${\mathrm{WO}}_{3}$/${\mathrm{CuWO}}_{4}$	${\mathrm{NO}}_{2}$	1	82 ^*a*^	0.05	93/28	[116]

## Data Availability

Data are contained within the article.

## Abbreviations

The following abbreviations are used in this manuscript:

2D	two-dimensional
AACVD	aerosol-assisted chemical vapor deposition
Ag NPs	silver nanoparticles
APTES	(3-aminopropyl)triethoxysilane
BP	black phosphorus
CdO	cadmium oxide
${\mathrm{CH}}_{2}$O	formaldehyde
${\mathrm{CH}}_{4}$	methane
CO	carbon monoxide
${\mathrm{CO}}_{2}$	carbon dioxide
CoPc	Cobalt Phthalocyanines
Co(tpfpp)ClO₄	Cobalt(II) 5,10,15,20-tetrakis(pentafluorophenyl)porphyrin perchlorate
CP	conducting polymers
${\mathrm{CsPbBr}}_{3}$	cesium lead bromide
CuPc	copper phthalocyanines
${\mathrm{CuWO}}_{4}$	copper tungstate
CVD	chemical vapor deposition
EDA	ethylenediamine
F-P	Fabry–Perot
GNF	graphite nanofiber
GO	graphene oxide
GQDs	graphene quantum dots
$H_{2}$	hydrogen
$H_{2}$S	hydrogen sulfide
HCHO	formaldehyde
HNs	hollow nanospheres
${\mathrm{In}}_{2}$$O_{3}$	indium oxide
InAG	${\mathrm{In}}_{2}$$O_{3}$ with Aminonaphthalene-1-sulfonic acid (ANS) and reduced graphene oxide
LED	light-emitting diode
LOD	limit of detection
MBs	microballs
${\mathrm{MoS}}_{2}$	molybdenum disulfide
${\mathrm{MoSe}}_{2}$	Molybdenum diselenide
${\mathrm{MoTe}}_{2}$	molybdenum ditelluride
MPs	metalloporphyrins
MPcs	metallophthalocyanines
MOSs	metal oxide semiconductors
MOs	metal oxides
MWIR	mid-wave infrared
$N_{2}$	nitrogen
$N_{2}$O	nitrous oxide
${\mathrm{NH}}_{3}$	ammonia
NiO	nickel oxide
${\mathrm{NO}}_{2}$	nitrogen dioxide
$O_{2}$	oxygen
$O_{3}$	ozone
PANI	polyaniline
PA	polyacetylene
Pd	palladium
PdO	palladium oxide
PDMS	polydimethylsiloxanes
POM	polyoxometalate
PPy	polypyrrole
ppm	parts per million
ppb	parts per billion
PTh	polythiophene
QDs	quantum dots
RF	radio frequency
rGO	reduced graphene oxide
RT	room temperature
S-${\mathrm{SnO}}_{2}$	sulfur-doped tin oxide
SMF	single-mode fiber
${\mathrm{SnO}}_{2}$	tin(IV) oxide
${\mathrm{SO}}_{2}$	sulfur dioxide
SPR	surface plasmon resonance
TEA	trimethylamine
${\mathrm{TiO}}_{2}$	titanium dioxide
TMDC	transition metal dichalcogenide
UV	ultraviolet
${\mathrm{WO}}_{3}$	tungsten trioxide
${\mathrm{WS}}_{2}$	tungsten disulfide
XPS	X-ray photoelectron spectroscopy
ZnO	zinc oxide
ZIF-8	zeolitic imidazole framework material
g-$C_{3}$$N_{4}$	graphitic carbon nitride
